# Genome-wide Regional Heritability Mapping Identifies a Locus Within the *TOX2* Gene Associated With Major Depressive Disorder

**DOI:** 10.1016/j.biopsych.2016.12.012

**Published:** 2017-09-01

**Authors:** Yanni Zeng, Pau Navarro, Masoud Shirali, David M. Howard, Mark J. Adams, Lynsey S. Hall, Toni-Kim Clarke, Pippa A. Thomson, Blair H. Smith, Alison Murray, Sandosh Padmanabhan, Caroline Hayward, Thibaud Boutin, Donald J. MacIntyre, Cathryn M. Lewis, Naomi R. Wray, Divya Mehta, Brenda W.J.H. Penninx, Yuri Milaneschi, Bernhard T. Baune, Tracy Air, Jouke-Jan Hottenga, Hamdi Mbarek, Enrique Castelao, Giorgio Pistis, Thomas G. Schulze, Fabian Streit, Andreas J. Forstner, Enda M. Byrne, Nicholas G. Martin, Gerome Breen, Bertram Müller-Myhsok, Susanne Lucae, Stefan Kloiber, Enrico Domenici, Major Depressive Disorder Working Group of the Psychiatric Genomics Consortium, Ian J. Deary, David J. Porteous, Chris S. Haley, Andrew M. McIntosh

**Affiliations:** a Division of Psychiatry, University of Edinburgh, Edinburgh; b Medical Research Council Human Genetics Unit, University of Edinburgh, Edinburgh; c Centre for Genomic and Experimental Medicine, University of Edinburgh, Edinburgh; d Generation Scotland, Centre for Genomic and Experimental Medicine, Institute of Genetics and Molecular Medicine, University of Edinburgh, Edinburgh; e Centre for Cognitive Ageing and Cognitive Epidemiology, University of Edinburgh, Edinburgh; f Department of Psychology, University of Edinburgh, Edinburgh; g The Roslin Institute and Royal (Dick) School of Veterinary Sciences, University of Edinburgh, Edinburgh; h Division of Population Health Sciences, University of Dundee, Dundee; i Division of Applied Health Sciences, University of Aberdeen, Aberdeen; j Institute of Cardiovascular and Medical Sciences, University of Glasgow, Glasgow; k MRC Social, Genetic, and Developmental Psychiatry Centre, Institute of Psychiatry, Psychology, and Neuroscience, King’s College London, London, United Kingdom; l Queensland Brain Institute, University of Queensland, St. Lucia, Queensland; m School of Psychology, University of Queensland, St. Lucia, Queensland; n Discipline of Psychiatry, University of Adelaide, Adelaide, Australia; o Department of Psychiatry, VU University Medical Center, Amsterdam, The Netherlands; p Department of Biological Psychology, VU University, Amsterdam, The Netherlands; q Department of Psychiatry, Lausanne University Hospital, Lausanne, Switzerland; r Institute of Psychiatric Phenomics and Genomics, Ludwig-Maximilians-University, Munich Cluster for Systems Neurology, Munich; s Max Planck Institute of Psychiatry, Munich Cluster for Systems Neurology, Munich; t Department of Psychiatry and Psychotherapy, University Medical Center, Georg-August-University, Göttingen; u Department of Genetic Epidemiology in Psychiatry, Central Institute of Mental Health, Medical Faculty Mannheim, University of Heidelberg, Heidelberg; v Department of Genetic Epidemiology in Psychiatry, Medical Faculty Mannheim, Central Institute of Mental Health, University of Heidelberg, Mannheim; w Institute of Human Genetics, Life and Brain Center, University of Bonn, Bonn, Germany; x Department of Genomics, Life and Brain Center, University of Bonn, Bonn, Germany; Y Laboratory of Neurogenomic Biomarkers, Centre for Integrative Biology, University of Trento, Trento, Italy

**Keywords:** Genome-wide analyses, Haplotype block, HRHM, MDD, Regional heritability, *TOX2*

## Abstract

**Background:**

Major depressive disorder (MDD) is the second largest cause of global disease burden. It has an estimated heritability of 37%, but published genome-wide association studies have so far identified few risk loci. Haplotype-block-based regional heritability mapping (HRHM) estimates the localized genetic variance explained by common variants within haplotype blocks, integrating the effects of multiple variants, and may be more powerful for identifying MDD-associated genomic regions.

**Methods:**

We applied HRHM to Generation Scotland: The Scottish Family Health Study, a large family- and population-based Scottish cohort (*N* = 19,896). Single-single nucleotide polymorphism (SNP) and haplotype-based association tests were used to localize the association signal within the regions identified by HRHM. Functional prediction was used to investigate the effect of MDD-associated SNPs within the regions.

**Results:**

A haplotype block across a 24-kb region within the *TOX2* gene reached genome-wide significance in HRHM. Single-SNP- and haplotype-based association tests demonstrated that five of nine genotyped SNPs and two haplotypes within this block were significantly associated with MDD. The expression of *TOX2* and a brain-specific long noncoding RNA RP1-269M15.3 in frontal cortex and nucleus accumbens basal ganglia, respectively, were significantly regulated by MDD-associated SNPs within this region. Both the regional heritability and single-SNP associations within this block were replicated in the UK–Ireland group of the most recent release of the Psychiatric Genomics Consortium (PGC), the PGC2–MDD (Major Depression Dataset). The SNP association was also replicated in a depressive symptom sample that shares some individuals with the PGC2–MDD.

**Conclusions:**

This study highlights the value of HRHM for MDD and provides an important target within *TOX2* for further functional studies.

Major depressive disorder (MDD) is ranked as the second leading contributor to the global disease burden in terms of years lived with disability (1). The narrow sense heritability of MDD has been estimated to be 37% by twin studies (2), suggesting a substantial contribution from genetic factors. In efforts to identify specific genetic risk factors for MDD, family-based linkage studies have identified several significant peaks in certain families, but the findings have been inconsistent (3). Genome-wide association studies (GWASs) of unrelated participants have successfully identified hundreds of loci associated with other psychiatric disorders (4), but for MDD only four genome-wide significant and replicable loci have been identified by two large GWASs: one on a refined MDD phenotype for Chinese women and one on self-report-based depression using less intensive phenotyping in a much larger European sample (5, 6, 7).

Several factors may be responsible for the comparatively sparse GWAS results in MDD. First, MDD is likely to have a highly polygenic genetic architecture where the disease risk is conferred by many causal variants of small effect (8, 9). Combined with the high prevalence of MDD (10) and the possible incomplete linkage disequilibrium (LD) between genotyped single nucleotide polymorphisms (SNPs) and causal SNPs, single-SNP-based genome-wide association tests may have insufficient power to detect individual causal variants (11). Second, clinical heterogeneity has been shown in MDD between populations (6, 12), and this may lead to difficulties in identifying causal variants across cohorts (13). Whereas GWAS sample sizes for MDD are increasing and efforts to refine the MDD phenotype are in progress (5, 7), alternative methodologies for detecting the signal arising from causal variants within and across families may also be productive.

Regional heritability mapping (RHM) is a method used to identify small genomic regions accounting for a significant proportion of the phenotypic variance in a trait of interest (14). In contrast to single-SNP-based tests, RHM integrates effects from multiple SNPs by using a regional genetic relationship matrix estimated from SNPs within a region. The matrix is constructed for each region defined by a sliding window across the genome and is then used to estimate the variance explained by the variants within the region in a linear mixed model (14). The major advantage of RHM is that the regional genetic relationship matrices not only tag the effect of genotyped variants but also measure the effect of ungenotyped and rare variants, including those associated with the SNPs but with individual effects too small to be detected by GWASs (14, 15). Previous studies have shown that RHM has greater power to detect rare variants and multiple alleles in regions where GWASs provided null findings (15, 16, 17). In 2014, Shirali *et al.* developed a haplotype-block-based RHM (HRHM) method as an improved version of RHM. HRHM uses haplotype blocks as the unit of mapping; therefore, the identified blocks have less complex local LD structures (18).

In this study, we applied HRHM to a homogeneous sample of approximately 20,000 Scottish participants containing both closely and distantly related subjects with genome-wide genotyping data and a standardized structured clinical MDD diagnosis (19). We sought to identify genomic regions conferring risk for MDD, which were then further explored using single-SNP- and haplotype-based association tests. We then examined the functional effects of the MDD-associated SNPs within the identified block. Finally, replication analyses were performed in independent samples for both the regional heritability and SNP association results.

## Methods and Materials

The Tayside Research Ethics Committee (reference 05/S1401/89) provided ethical approval for the study. Participants all gave written consent after having an opportunity to discuss the project and before any data or samples were collected.

### Datasets

#### Discovery Sample: Generation Scotland: The Scottish Family Health Study

Generation Scotland: The Scottish Family Health Study (GS:SFHS) contains 21,387 subjects (*n*_male_ = 8772, *n*_female_ = 12,615; age_mean_ = 47.2 years, SD = 15.1) who were recruited from the registers of collaborating general practices in Glasgow, Tayside, Ayrshire, Arran, and Northeast regions of Scotland, United Kingdom. At least one first-degree relative aged 18 years or over was required to be identified for each participant (19, 20). A structured clinical interview was used for the diagnosis of lifetime DSM-IV mood disorders (21, 22). Details of MDD diagnosis, genotyping, quality control, and imputation methods are described in the Supplement. In total, 561,125 genotyped and 8,642,105 postimputation autosomal SNPs that passed quality control criteria were available for 19,896 participants (2659 MDD cases and 17,237 control subjects) for subsequent analyses.

#### Replication Sample 1: UK Biobank

Data used in this study were provided as part of the UK Biobank project (reference no. 4844). Details for genotyping, quality control, imputation, and phenotyping are described in the Supplement. In brief, genotyping data were available for 152,729 UK Biobank participants recruited in the United Kingdom (23). The probable MDD phenotype was created based on the putative MDD definition established in Smith *et al.* using responses to a touchscreen questionnaire (24), from self-report information, and from inpatient records via linkage to hospital episode data (see Supplement). After quality control and removing subjects who were in both the GS:SFHS and UK Biobank datasets, and one of each pair of close relatives (relatedness >0.05) of GS:SFHS participants or the remaining UK Biobank participants, 1,198,327 SNPs for 24,015 subjects with the putative MDD phenotype available (8143 cases and 15,872 control subjects) remained in downstream analyses.

#### Replication Sample 2: Psychiatric Genomics Consortium Major Depression Dataset

The Psychiatric Genomics Consortium (PGC) provided individual genotypes (best guess) of imputed SNPs for participants from 22 cohorts in the PGC Major Depression Dataset (PGC2–MDD) (Supplemental Table S1). All cases met DSM-IV criteria for life MDD; the majority of them were ascertained clinically. Most control samples were screened, and participants with lifetime MDD were removed (Supplemental Table S1). Details for genotyping, quality control, imputation, and phenotyping are described in the Supplement. After quality control and removing subjects who overlapped with the GS:SFHS and UK Biobank datasets, 32,554 subjects of European ancestry (13,261 cases and 19,293 control subjects) were used in downstream analysis. Consistent with earlier work (25, 26), we grouped the 22 cohorts into 7 groups based on the country of ancestor information for regional heritability analysis (Supplemental Table S1).

#### Replication Sample 3: Depressive Symptom Datasets

The depressive symptom (DS) sample contains overlapping individuals with replication samples 1 and 2. Okbay *et al.* carried out a GWAS meta-analysis (*N* = 180,866) on three samples using depressive symptoms as the trait of interest (27). The ascertained MDD diagnosis information was available for two samples: PGC1–MDD (*n*_cases_ = 9240, *n*_controls_ = 9519) and the Resource for Genetic Epidemiology Research on Aging (*n*_cases_ = 7231, *n*_controls_ = 49,316) (27). For the third sample, UK Biobank (*N* = 105,739), a continuous phenotype measuring the severity of depressive symptom had been created and used in the meta-analysis (27). Although this sample overlapped with the PGC2–MDD and UK Biobank samples, it provided results based on a nondiagnostic quantitative measure of depressive symptoms and involved another large cohort, the Resource for Genetic Epidemiology Research on Aging (27).

### Genome-wide HRHM

RHM is a method for detecting localized genomic regions where genetic variants contribute significantly to the variation of phenotype of interest (14). As an improved version of RHM, HRHM divides the genome into haplotype blocks based on the recombination hotspots in the genome (18). Details of HRHM are described in the Supplement. In brief, in GS:SFHS, the genotyped SNPs were mapped to 49,637 haplotype blocks across the genome and the regional heritability was estimated and tested for each of the haplotype blocks. A standard “two-GRM” model incorporates two genomic relationship matrices (GRMs): a regional genomic relationship matrix (rGRM) estimated from SNPs in the haplotype block and a complement genomic relationship matrix (cGRM) estimated from all SNPs that are not included in the haplotype block. These GRMs were jointly fitted as random effects in linear mixed models. Covariates fitted as fixed effects include age, age^2^, sex, and 20 principal components. A log likelihood ratio test (LRT) is applied to test the significance of random effect represented in an rGRM by comparing a model with both a cGRM and an rGRM fitted against a model including the cGRM but without an rGRM fitted. The genome-wide significance threshold for *p* values from the LRT is 1.01 × 10^–6^ (*N*_Bonferroni_ = 49,637). This two-GRM model, while providing an unbiased estimate of regional heritability, was highly computationally demanding. To improve the calculation efficiency, a preadjustment strategy was applied in the genome-wide HRHM (see Supplement). For haplotype blocks that exceeded the genome-wide significant threshold, we retested the block using the two-GRM model to provide an accurate estimation of regional heritability in the target block. All the analyses were performed in REACTA (14, 28). According to the GCTA-GREML Power Calculator, this study is well powered for the genomic-relatedness-based restricted maximum-likelihood-based SNP heritability analysis (99.88%) (29).

### Localized Association Tests for the Significant Haplotype Block Identified by HRHM in GS:SFHS

HRHM identified a significant block chr20:42555671–42579473, and we performed a series of association tests to localize the association signals within this block in GS:SFHS.

#### Single-SNP-Based Association Test for Common SNPs Within the Identified Haplotype Block

Association tests were performed on genotyped and imputed common SNPs located in the significant haplotype block chr20:42555671–42579473 using GCTA–MLMA (mixed linear model-based association analysis) (30). The SNP effect was tested as a fixed effect; other covariates included age, age^2^, sex, and 20 principal components. To prevent the estimates of SNP effects from being confounded by the polygenic component and family structure, cGRM and cGRM_kin_ were fitted simultaneously as random effects in the model (31). cGRM (complement-SNP-set GRM) was the genomic relationship created matrix using all of the genotyped SNPs, excluding the SNPs in the hit block; cGRM_kin_ was the kinship relationship matrix (representing pedigree-associated genetic variation). cGRM_kin_ was created by setting elements in cGRM that were less than or equal to 0.05 to 0 (31). The estimated fixed effect (on the linear scale) was transformed to logit and liability scale using Taylor series approximation (32). Bonferroni multiple testing correction was performed for the *p* values for each SNP.

#### Single-Haplotype-Based Association Test

Single-haplotype-based association tests were performed for the common haplotypes (frequency ≥ 0.01) derived from the nine genotyped common SNPs located in the significant haplotype block chr20:42555671–42579473 using GCTA–MLMA (30) for the full dataset and an unrelated dataset and using famLBL (family-triad-based logistic Bayesian Lasso) (33) for a subset consisting of case–parent trios in GS:SFHS. Details of the single-haplotype-based association test are described in the Supplement.

### Functional Effects of MDD-Associated SNPs in the Significant Block

The significant haplotype block chr20:42555671–42579473 is located in the intron region and a proportion of an adjacent exon of gene *TOX2*. To investigate the potential functional effects from variants within this block, we imputed the nine genotyped SNPs within this block to 53 common SNPs based on Haplotype Reference Consortium reference; all of them are noncoding SNPs. We performed the single-SNP-based association test for each of them with MDD using GCTA–MLMA (the same method for genotyped SNPs). This identified 38 imputed SNPs significantly associated with MDD. We then examined the functional role of the 38 SNPs using the following functional annotation tools and analyses: the potential to affect the binding of transcription factors in RegulomeDB (34), Genome Wide Annotation of Variants (GWAVA), Genomic Evolutionary Rate Profiling (GERP) (35), brain-tissue-specific allelic effect on gene expression (expression quantitative trait loci [eQTL] analysis) based on GTEx and BRAINEAC databases, and brain-tissue-specific allelic effect on DNA methylation in CpG loci (methylation quantitative trait loci [meQTL] analysis). Details of these tools and analyses are described in the Supplement.

### Replication Analysis

#### Regional Heritability in the Significant Block Identified in GS:SFHS

Individual genotypes in UK Biobank and PGC2–MDD (22 cohorts) were used to estimate the regional heritability of the target haplotype block in the two samples. The two-GRM model (rGRM + cGRM) was applied to provide accurate estimates. For PGC2–MDD, the regional heritability was estimated for each of the 7 groups defined based on country of ancestor (Supplemental Table S1) as well as for the combined dataset.

#### Single-SNP-Based Association Test for the Five Significant SNPs (Genotyped) Within the Significant Block Identified in GS:SFHS

For UK Biobank, the single-SNP-based association tests were performed using a logistic model in PLINK (36). Covariates included age, sex, center, batch, and 15 principal components provided by UK Biobank. For PGC2–MDD, the association test was performed using a logistic model for each individual cohort. Covariates include sex and 20 principal components (the age variable was not yet available for the full dataset at the time of this study). Meta-analysis was performed across all cohorts in each group to generate group-level association statistics. The meta-analysis was performed using the “metagen” function in the R package “meta”. For the DS sample the GWAS summary statistics were downloaded from the website of the Social Science Genetic Association Consortium (http://www.thessgac.org/#!data/kuzq8).

## Results

**Figure 1:**
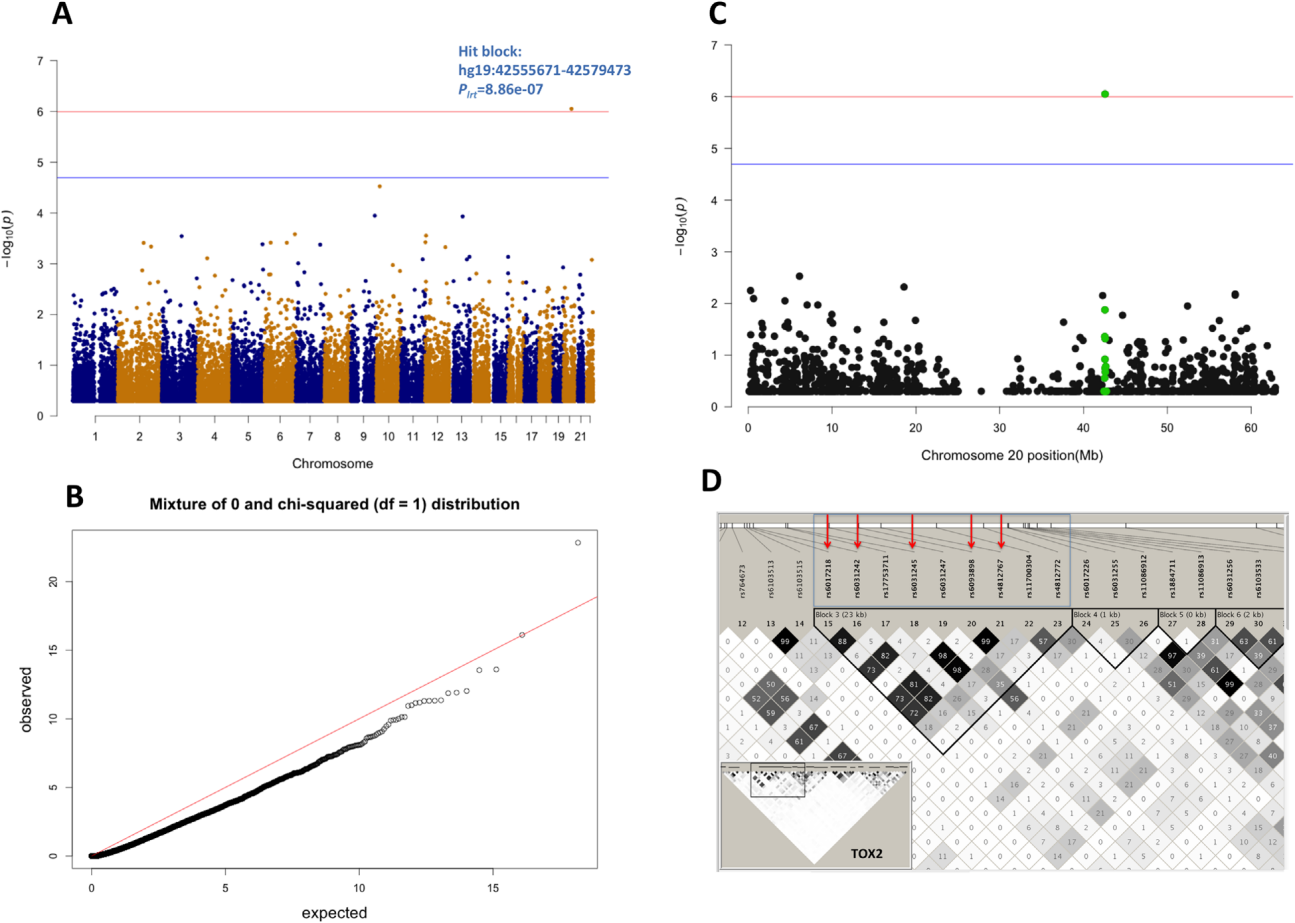
Genome-wide haplotype-block-based regional heritability mapping results on major depressive disorder in Generation Scotland: The Scottish Family Health Study (GS:SFHS). **(A)** Manhattan plot. Each point represents a haplotype block. The location of the point is the mid-position of the haplotype block. **(B)** A quantile-quantile plot for the likelihood ratio test (LRT). The LRT statistics are distributed as a mixture of 0 and chi-squared (*df* = 1) distribution. **(C)** Zoom-in region of the hit haplotype block region in chromosome 20. **(D)** Linkage disequilibrium (LD) structure within the hit haplotype block in GS:SFHS. The block is located in gene *TOX2;* it contains nine genotyped common SNPs (blue boxes), and five of them are in high LD (red arrows) in GS:SFHS.

Genome-wide HRHM was carried out for 49,637 haplotype blocks using 561,125 genotyped common SNPs in GS:SFHS for MDD (*n*_case_ = 2659, *n*_control_ = 17,237). The regional heritability from each haplotype block was tested using a preadjusted GRM strategy in the linear mixed model. The Manhattan plot and quantile-quantile plot for the LRT are shown in Figure 1. One haplotype block covering a 24-kb region in the intron region and a proportion of an adjacent exon of gene *TOX2* exceeded the genome-wide significant threshold (*p*_Bonf_threshold_ = 1.01 × 10^–6^): hg19:chromosome20:42555671–42579473 (*p*_lrt_ = 8.86 × 10^–7^) (Figure 1). The two-GRM model confirmed the significance of this haplotype block (*p*_lrt_ = 5.6 × 10^–7^), and the regional heritability ($h_{\;g}^{2}$) was estimated to be 0.008 (0.006). The regional heritability of this block was more significant in female MDD ($h_{\;g}^{2}$ = 0.009, SE = 0.007, *p*_lrt_ = 5.64 × 10^–5^, *n*_case_ = 1893, *n*_control_ = 9818) than in male MDD ($h_{\;g}^{2}=$ 0.003, SE = 0.004, *p*_lrt_ = .02, *n*_case_ = 765, *n*_control_ = 7420).

We further performed a series of association tests to disentangle the signal detected by HRHM in the significant block. Using the single-SNP-based association test, five of the nine genotyped common SNPs within the hit block were significantly associated with MDD (Table 1 and Supplemental Table S2). The five significant SNPs were in high LD with each other (Figure 1D), and their minor alleles showed a consistent negative effect on the risk of MDD, with the odds ratio ranging from 0.785 to 0.833 (Table 1). Haplotype-based association tests for haplotypes derived from the nine SNPs showed that two of the seven common haplotypes (frequency ≥ 0.01) were associated with MDD. One of these haplotypes contains the minor (protective) alleles of the five single-SNP-level significant SNPs, and one contains the major (risk) alleles. The size and direction of the effects of the two haplotypes were consistent with those estimated from the single-SNP-based tests (odds ratio of 0.792 for the protective haplotype and 1.232 for the risk haplotype) (Table 2). Additional association tests on subdatasets (unrelated and case–parent trio) showed that the risk haplotype was significantly associated with MDD in the unrelated dataset (Supplemental Table S3), whereas the protective haplotype was significant in the case–parent trio dataset (Supplemental Table S4).

**Table 1:** Single-SNP-Based Association Test Results for Five MDD-Associated SNPs in Discovery and Replication Samples

SNP Information					Discovery: GS:SFHS				Replication 1: UK Biobank				Replication 2: PGC2–MDD (UK–Ireland)				Replication 3: DS		
rs ID	Chr	Pos	A1	A2	OR	logOR	SE (logOR)	*p*	OR	logOR	SE (logOR)	*p*	OR	logOR	SE (logOR)	*p*	Beta	SE	*p*
rs6017218	20	42555737	G(C)	T(A)	0.833	−0.183	0.041	2.44E-04	0.947	−0.055	0.030	.068	0.842	−0.172	0.068	.011	−0.013	0.005	.007
rs6031242	20	42556096	G(C)	A(T)	0.832	−0.184	0.043	4.36E-04	0.948	−0.054	0.032	.090	0.859	−0.153	0.071	.032	−0.012	0.005	.018
rs6031245	20	42559531	T(A)	C(G)	0.783	−0.244	0.045	2.30E-05	0.958	−0.043	0.035	.225	0.843	−0.171	0.076	.024	−0.015	0.006	.011
rs6093898	20	42566577	G(C)	A(T)	0.783	−0.245	0.045	2.03E-05	0.958	−0.043	0.035	.222	0.848	−0.165	0.075	.028	−0.016	0.006	.006
rs4812767	20	42568829	T(A)	C(G)	0.785	−0.242	0.045	2.57E-05	0.961	−0.040	0.035	.253	0.840	−0.174	0.075	.021	−0.016	0.006	.006

Chr, chromosome; DS, Depressive Symptom; GS:SFHS, Generation Scotland: The Scottish Family Health Study; MDD, major depressive disorder; OR, odds ratio; PGC2–MDD, Psychiatric Genomics Consortium–Major Depression Dataset; Pos, position; SNP, single nucleotide polymorphism.

**Table 2:** Haplotype-Based Association Test Results for Common Haplotypes Derived From the Nine Genotyped Common SNPs in GS:SFHS

Haplotype	Frequency	Beta (Linear)	SE (Beta [Linear])	OR	logOR	SE (logOR)	*p*	Adjusted *p*
TAGCGACCT	0.120	0.026	0.005	1.232	0.209	0.058	2.47E-06	1.73E-05^a^
GGGTGGTCC	0.094	−0.024	0.006	0.792	−0.233	0.046	5.77E-05	4.04E-04^a^
TAGCAACCT	0.118	−0.010	0.005	0.911	−0.093	0.045	6.10E-02	4.27E-01
TAGCGACTC	0.311	0.006	0.004	1.052	0.051	0.035	1.24E-01	8.71E-01
GAGCAACCT	0.012	−0.012	0.016	0.897	−0.109	0.131	4.60E-01	1.00E+00
TAGCAACCC	0.015	−0.010	0.014	0.916	−0.088	0.120	5.05E-01	1.00E+00
TATCGACTC	0.304	−0.002	0.004	0.980	−0.020	0.033	5.59E-01	1.00E+00

Adjusted *p:* Bonferroni method adjusted *p* values.

GS:SFHS, Generation Scotland: The Scottish Family Health Study; OR, odds ratio; SNP, single nucleotide polymorphism.

a Significant results.

**Figure 2:**
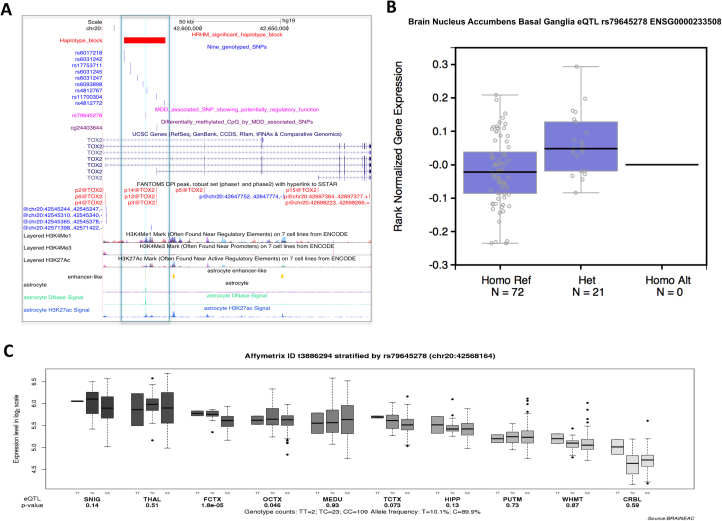
Functional prediction of the hit haplotype block. **(A)** Functional annotation of the hit block. The hit haplotype block (red bar on the left top showing the block and blue bars showing the genotype single nucleotide polymorphisms [SNPs] in Generation Scotland: The Scottish Family Health Study [GS:SFHS]) is located in the intron region and a proportion of an adjacent exon of gene *TOX2,* overlapped with Fantom5 enhancers and transcription start sites, and regulatory-relevant histone modification peaks (H3K27Ac and H3K4Me1). Within the block, 38 imputed SNPs were associated with major depressive disorder (MDD), using SNP rs79645278 (pink) as an example. This SNP is located in the peak of active enhancer in astrocyte (highlighted with blue line). **(B, C)** Boxplots showing tissue-specific effect from SNPs that are both associated with MDD in GS:SFHS and gene expression, using SNP rs79645278 as an example. **(B)** The minor allele of rs79645278 upregulates the expression of a long noncoding RNA RP1-269M15.3 in the tissue nucleus accumbens basal ganglia. **(C)** The minor allele of rs79645278 upregulates the expression of gene *TOX2* in the frontal cortex (FCTX). CRBL, cerebellar cortex; eQTL, expression quantitative trait loci; HIPP, hippocampus; MEDU, medulla (specifically inferior olivary nucleus); OCTX, occipital cortex (specifically primary visual cortex); PUTM, putamen; SNIG, substantia nigra; THAL, thalamus; TCTX, temporal cortex; WHMT, intralobular white matter.

The significant block overlapped with an enhancer active in multiple tissues and cell lines, including astrocytes (Figure 2A) (37), and multiple alternative transcription start sites (TSSs) including a TSS primarily expressed in the thalamus (the TSS labeled as “p3@TOX2” in Figure 2A) (37), suggesting a potential regulatory role. To link the association signal from single variants with the potentially functional effects of those variants on disease-relevant biological processes, we identified 38 imputed SNPs in the target block significantly associated with MDD (Supplemental Table S5) and predicted their potentially regulatory function using multiple predictors and statistics of noncoding DNA function, including the likelihood of affecting transcription factor binding, multiple genome-wide properties, evolutionary conservation, and the *cis* effect on gene expression of genes within a distance of 1 MB and on DNA methylation. Among the 38 SNPs, 2 were annotated to be “likely to affect TF binding” (score = 2b) by RegulomeDB, 5 obtained a GWAVA–TSS score ≥ 0.5 (suggesting “functional”), and 5 obtained a GERP score > 2 (suggesting “constrained”) (Supplemental Table S6). Tissue-specific SNP-*cis*-gene expression (*cis*-eQTL) analyses were performed for the 38 SNPs using 11 brain tissues from GTEx and 10 brain tissues from BRAINEAC. The results from GTEx showed that the genotypes of 30 of the 38 SNPs significantly stratify the expression of gene RP1-269M15.3 (long noncoding RNA [LncRNA]) in the tissue nucleus accumbens basal ganglia, with the minor alleles significantly upregulating the RNA expression level (Supplemental Table S7) (Figure 2B). The results from BRAINEAC suggested that all 38 SNPs significantly stratify the expression of gene *TOX2* in the frontal cortex (minor allele induces upregulation) (Figure 2C) and gene C20orf62 (LncRNA) (minor allele induces downregulation) in the cerebellar cortex (Supplemental Tables S8 and S9). The results from meQTL analysis suggested that 30 of the 38 SNPs are significant meQTL SNPs in the frontal cortex and that particularly 19 of them significantly stratify DNA methylation of a CpG locus cg24403644 (minor allele induces hypomethylation) (Supplemental Table S10). The locus cg24403644 is located in a cluster of TSSs in *TOX2* (Figure 2) and shows differential methylation between human fetal and postnatal lifetime in the frontal cortex and during fetal brain development (38, 39). Among significant SNPs in the *cis*-eQTL and *cis*-meQTL analyses, rs79645278 was located in the peak of active enhancer (in astrocytes and other cell lines) and was predicted to be “likely to affect TF binding” (2b) in RegulomeDB, having a GWAVA–TSS score of 0.5 and a GERP score of 2.31 (Figure 2A–C and Supplemental Table S6).

**Figure 3:**
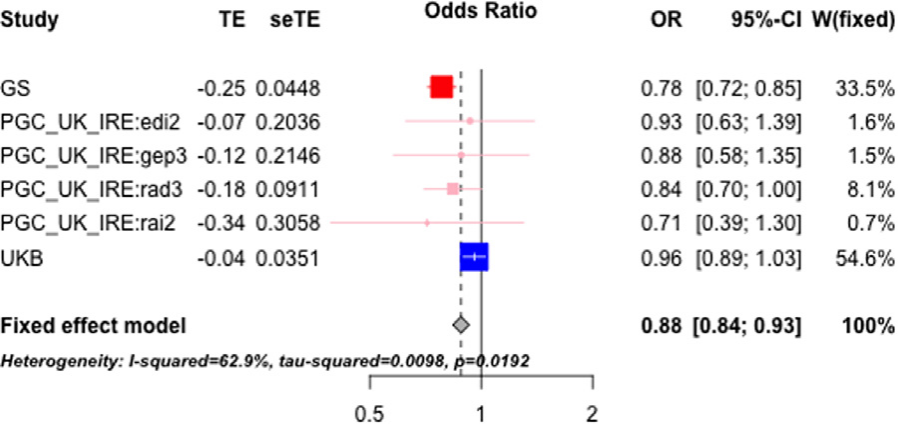
Forest plot showing meta-analysis for single-single nucleotide polymorphism (SNP)-based association test on Generation Scotland: The Scottish Family Health Study and all UK–Ireland replication samples (four Psychiatric Genomics Consortium–Major Depression Dataset [PGC2–MDD] cohorts and UK Biobank), using SNP rs6093898 as an example. CI, confidence interval; OR, odds ratio; seTE, standard error of the estimate; TE, estimate of effect size; W, weight of individual studies.

The regional heritability detected in the hit block was replicated in the UK–Ireland group in PGC2–MDD with nominal significance (*p*_lrt_ = .049, $h_{\;g}^{2}$= 0.001, SE = 0.001), whereas it was not significant in other groups in PGC2–MDD and UK Biobank (Supplemental Table S11). The single-SNP-based association test for the five significant SNPs (genotyped) in this block identified in GS:SFHS showed that all five were replicated in the DS sample; all five were also replicated in the UK–Ireland group in PGC2–MDD (Table 1). Results for individual cohorts are shown in Supplemental Table S12 and Supplemental Figure S1 but not in other PGC2–MDD groups or in the meta-analyzed combined PGC2–MDD sample (Supplemental Table S13); none of the five SNPs were replicated in the UK Biobank sample, but all showed the same consistent direction of effect as that reported in the discovery sample (Table 1 and Supplemental Figure S1). Meta-analysis using all independent UK–Ireland replication samples (UK Biobank and four cohorts in PGC2–MDD and UK–Ireland) showed that all five SNPs reached nominal significance (Supplemental Table S13), consistent sign with GS:SFHS as shown in Figure 3, using SNP rs6093898 as an example.

## Discussion

The current study used a combination of genome-wide HRHM, localized association tests, and functional prediction to identify candidate genomic regions associated with MDD. Using the large Scottish cohort GS:SFHS, a genome-wide significant haplotype block located in gene *TOX2* was identified by HRHM as a risk region for MDD. Association tests using both single SNPs and haplotypes within this block highlighted candidates contributing genetic variants for MDD. Replication analyses showed that the regional heritability in this block was nominally significant in the UK–Ireland groups in PGC2–MDD. The SNP-level association signals within the hit block were replicated in the UK–Ireland group in PGC2–MDD and a study of DS that has overlapping subjects from PGC2–MDD and UK Biobank.

As shown in this study, compared with single-SNP-based genome-wide association methods, HRHM provided the following advantages. First, a smaller number of tests were performed; therefore, a less stringent threshold of genome-wide significance was applied. Second, haplotype blocks rather than single SNPs were the unit of mapping; therefore, these are relatively less dependent on the density of the genotype arrays and do not require the same SNPs to be typed or imputed in replication studies. Third, HRHM applied a linear mixed model accounting for both polygenic component and family structure, and it can be applied to both population and family data. Fourth, because haplotype blocks were used as the unit of mapping, the identified locus has a less complex LD structure (Figure 1D), which will benefit the downstream identification of candidate variants.

To date, published GWASs have mapped associated variants to very few genes for MDD (*LHPP, SIRT1, TMEM161B–MEF2C,* and *NEGR1*) (5, 7). In this study, the identified haplotype block was located in gene *TOX2* (TOX high mobility group box family member 2, also known as *GCX1*), indicating a new candidate gene for MDD. *TOX2* is a putative transcriptional activator involved in the hypothalamo–pituitary–gonadal system (40) and is located in a large genomic region that has been previously reported as associated with depression symptoms in psychotic illness (41, 42). The same locus has also been weakly associated with conduct disorder in a previous study (43). Using available databases, we found that convergent evidence from TSS by Fantom5 annotation (Figure 2A), histone modification markers and DNase peaks representing active enhancers by ENCODE annotation (Figure 2A), and transcription factor binding prediction by RegulomeDB (Supplemental Table S6) suggested a regulatory function of this block. To test for the potential effects of the variants within the block on gene expression, we performed brain-tissue-specific *cis*-QTL analysis for SNPs significantly associated with MDD within the block. The expression of an LncRNA RP1-269M15.3 was significantly upregulated by the minor alleles (minor alleles are protective to MDD, as shown in Table 1 and Supplemental Table S5) of candidate SNPs within the block in nucleus accumbens, a tissue having been previously implicated in MDD (44). RP1-269M15.3 was a multiexon LncRNA with a multispecies conserved region (Supplemental Figure S2A) and was expressed specifically only in brain tissues (Supplemental Figure S2B) and therefore is of potential function in brain tissues. Similarly, the expression of gene *TOX2* was significantly upregulated by the minor alleles of candidate SNPs in the frontal cortex, a relevant tissue of MDD as well (45). The regulatory effect of MDD-associated SNPs in gene *TOX2* in the frontal cortex is further supported by the meQTL analysis on the same tissue. Combined with the fact that all 19 SNPs are both meQTL and eQTL SNPs for gene *TOX2* in the frontal cortex and the fact that hypomethylation has been previously suggested to be correlated with up-regulation of gene expression (46), consistent evidence from both methylation and gene expression data indicated that the minor alleles (protective) of MDD-associated SNPs upregulate the gene expression of *TOX2* in the frontal cortex (Supplemental Tables S8 and S10). Interestingly, the brain-specific expressions of both RP1-269M15.3 and *TOX2* were highly correlated (*r* ≥ .70) with a number of depression-related genes (e.g., *LRFN5, GRM7, CRH*) (47, 48) in brain development (http://brainspan.org) (Supplemental Tables S14 and S15), suggesting that the expression networks involving those genes were potential targets of the effects from candidate variants. These results are consistent with a previous study suggesting an overrepresentation of MDD GWAS significant loci in central nervous system expression and the regulation of gene expression in the central nervous system during development (7).

The regional heritability in the identified block was nominally significant only in the UK–Ireland group of PGC2–MDD. The five significant genotyped SNPs within the block identified in GS:SFHS were replicated in the DS sample and in the UK–Ireland group in PGC2–MDD. The UK Biobank sample failed to replicate any of them, although it showed a consistent sign of effect. Those results are likely attributable to the phenotyping differences [diagnosed MDD in GS:SFHS, mostly diagnosed MDD in PGC (49), putative MDD in UK Biobank, and depressive symptom in DS] and the clinical heterogeneity within MDD across PGC2–MDD groups as shown in Supplemental Table S10 (12). Notably, UK–Ireland, which shows the most consistent replication results, is from the same country/region as GS:SFHS, so its cohorts are likely to have a similar local genomic recombination pattern and LD structure with GS:SFHS and potentially carry alleles not common in other European cohorts, which may explain the better replication result from this group (Figure 3 and Supplemental Figure S1).

There are, however, several limitations in the current study. First, the readjustment strategy applied to genome-wide HRHM; while it reduced the computational burden, it was potentially excessively conservative in reporting true associations (observed LRT statistics were depleted from expectation, as shown in Figure 1D), which consequently reduced the power of HRHM (50). Second, phenotypic difference among discovery and replication samples impeded the complete replication of findings across all samples. UK Biobank samples are also from the same country/region as GS:SFHS, as is the UK–Ireland group of PGC2–MDD, but currently UK Biobank samples have only putative MDD information available for a small subset of genotyped participants. Ongoing clinical assessment of MDD and the genotyping work on these samples will potentially provide more power to the replication analysis for our findings in future data releases.

### Conclusions

The current study showed the first application of genome-wide HRHM to a psychiatric disorder. A genome-wide significant region was identified by HRHM, and the contributing genetic effect was localized to variants and haplotypes within the block. The results were partly replicated in two independent samples. Functional prediction and *cis*-eQTL analyses suggested that the genotype of associated variants within the block stratified the gene expression of a potentially functional LncRNA RP1-269M15.3 and gene *TOX2* in MDD-relevant brain tissues, which should be explored in further studies.
