# Supplementary material for: Genome-wide Regional Heritability Mapping Identifies a Locus Within the TOX2 Gene Associated With Major Depressive Disorder
Source: Biol Psychiatry. 2017 Sep 1;82(5):312–21. doi: 10.1016/j.biopsych.2016.12.012 (PMC5553996; doi:10.1016/j.biopsych.2016.12.012)
Supplement: Supplementary file 1 — Supplementary material [file mmc1.pdf]

Genome-wide Regional Heritability Mapping Identifies a Locus Within the *TOX2* Gene Associated With Major Depressive Disorder

Supplementary Information

CONTENTS

Supplemental Methods

Genotyping, quality control, imputation and phenotyping details in GS:SFHS dataset

Genotyping, quality control, imputation and phenotyping details in UK Biobank dataset

Genotyping, quality control, imputation and phenotyping details in PGC2-MDD dataset

Genome-wide haplotype-block-based regional heritability mapping

Single-haplotype-based association test

Functional annotation tools and analyses for MDD-associated SNPs

Table S1. Individual cohort and grouping information for 22 cohorts in PGC2-MDD

Table S2. Single-SNP based association test results for nine genotyped SNPs in the hit haplotype block in GS:SFHS

Table S3. Haplotype-based association test results for common haplotypes derived from nine common SNPs in hit block on unrelated individual dataset of GS:SFHS

Table S4. Haplotype-based association test results for common haplotypes derived from nine common SNPs in hit block on case-parents-trio of GS:SFHS

Table S5. Results of single-SNP-based association test on 53 imputed common SNPs in the hit block in GS:SFHS

Table S6. The functional prediction of 38 significant SNPs using regulomeDB score, GWAVA-Tss score and GERP score

Table S7. Significant results (Q value ≤ 0.05) from SNP-cis-gene-eQTL analysis using GTEX

Table S8. Significant results (Q value ≤ 0.05) from SNP-cis-gene-eQTL analysis on frontal cortex using BRAINEAC

Table S9. Significant results (Q value ≤ 0.05) from SNP-cis-gene-eQTL analysis on cerebellar cortex using BRAINEAC

Table S10. Significant results of meQTL SNPs for CpG locus cg24403644 (FDR ≤ 0.05) from SNP-cis-CpG DNA methylation analysis on frontal cortex

Table S11. Regional heritability estimates of the hit block in seven groups and the combined (22 cohorts) in PGC2-MDD and UK Biobank

Table S12. Single-SNP-based association tests for five SNPs in individual cohorts PGC2-MDD

Table S13. Meta-analysis results for five SNPs in groups and combined sample of PGC and all UK replication samples

Table S14. Genes showing similar expression patterns with RP1-269M15.3 (r≥0.7) in development brain tissues in BRAINSPAN

Table S15. Genes showing similar expression patterns with *TOX2* (r≥0.7) in development brain tissues in BRAINSPAN

Figure S1. Forest plots of meta-analysis of GS:SFHS, PGC2-MDD individual cohorts and UK Biobank for five SNPs

Figure S2. Functional annotation and gene expression patterns of RP1-269M15.3

Supplemental References

Supplemental Methods

Genotyping, quality control, imputation and phenotyping details in GS:SFHS dataset

Genotyping data were generated using the Illumina Human OmniExpressExome -8- v1.0 array (1). Details of genotyping are described elsewhere (2). Quality control (QC) of genotyped SNPs used inclusion thresholds: missing SNPs per individual ≤2%, SNP genotype call rate ≥98%, minor allele frequency (MAF) >1% and Hardy-Weinberg equilibrium P value > 1 x 10^{-6}. In total, 561,125 genotyped autosomal SNPs passed QC criteria and were used in the subsequent analyses. Haplotypes were identified using SHAPEIT for phasing with the option --duohmm which refines the phasing by pedigree information (3). Imputation was performed using the Sanger Imputation server (https://imputation.sanger.ac.uk/) (HRC). After removing imputed SNPs with info score <0.8 and MAF < 0.01, 8,642,105 imputed SNPs remained.

Diagnosis of MDD: A structured clinical interview was used for the diagnosis of lifetime DSM-IV mood disorders (SCID) (4,5). Participants screened positive for mental health problems by endorsing one or more screening questions: “Have you ever seen anybody for emotional or psychiatric problems?” or “Was there ever a time when you, or someone else, thought you should see someone because of the way you were feeling or acting?” (prevalence=21.7%). At the end of the screening questions, participants screening positive were invited to continue to an interview using the SCID modules for mood disorders (4). Participants were excluded from the study when: 1) they screened positive for mental health problems but refused to undergo the structured clinical interview (N=507) or 2) those who fulfilled criteria for bipolar disorder (N=76). This left 19,896 genotyped participants with 2,659 MDD cases and 17,237 controls for the downstream analysis.

Genotyping, quality control, imputation and phenotyping details in UK Biobank dataset

UK Biobank recruited around 500,000 people aged between 40-69 years in 2006-2010 across the United Kingdom (UK Biobank 2011a) (6).

Genotyping and imputation: genotyping was performed for 152,729 UK Biobank participants using the Affymetrix UK Biobank Axiom array (N=102,750) and the Affymetrix UK BiLEVE Axiom array (N=49,979). Imputed data were generated by UK Biobank using a modified version of SHAPEIT for phasing and imputation was carried out using IMPUTE2, based on 1000 Genomes Phase 3 and UK10K haplotype panels (3,7).

Additional QC was performed by removing SNPs with MAF < 0.01 and info score < 0.9. As the regional heritability analysis is highly computationally demanding, only those QC'ed imputed SNPs that could be mapped to the HapMap3 reference panel were used in downstream analyses (8). Additional filtering was performed for the genotyped subjects: 1) Non-white British participants were removed to reduce possible population stratification; this left 120,091 participants. 2) Subjects who were in both GS:SFHS and UK Biobank datasets were removed. 3) One of each pair of close relatives (relatedness > 0.05) of GS:SFHS participants or the remained UK Biobank participants were removed from the UK Biobank sample. This left 116,981 genotyped participants.

MDD phenotype: The probable MDD phenotype was created based on the putative MDD definition established in Smith et al (2013) using responses to a touchscreen questionnaire (UK Biobank 2011b) (9), from self-report information, and from inpatient records via linkage to hospital episode data. In detail, all controls were assessed to be absent of depressive symptoms (9) by satisfying the following criteria: 1) The answers are 'No' for the following touch screen questionnaire at recruitment: "Looking back over your life, have you ever had a time when you were feeling depressed or down for at least a whole week?", "Have you ever seen a psychiatrist for nerves, anxiety, tension or depression?", "Have you ever had a time when you were uninterested in things or unable to enjoy the things you used to for at least a whole week?". 2) No primary diagnosis of ICD-10 codes for mood disorders based on Hospital Episodes Data from UK bodies (English HES Data, Scottish Morbidity Register, Patient Episode Data). Cases satisfied the following criteria: 1) The answers of the following touchscreen questionnaire at recruitment are: 'Yes' for "Have you ever seen a psychiatrist for nerves, anxiety, tension or depression?"; either 'Yes' for "Looking back over your life, have you ever had a time when you were feeling depressed or down for at least a whole week?" and 'More than two weeks' for "How many weeks was the longest period when you were feeling depressed or down?" or 'Yes' for "Have you ever had a time when you were uninterested in things or unable to enjoy the things you used to for at least a whole week?" and 'More than two weeks' for "How many weeks was the longest period when you were uninterested in things or unable to enjoy the things you used to?". 2) Based on Hospital Episodes Data from UK bodies (English HES Data, Scottish Morbidity Register, Patient Episode Data), have a diagnosis of ICD-10 codes for mood disorders. Additional sample filtering procedures were applied on the basis of self-report information and linked health records. Participants satisfying following criteria were excluded in the sample: 1) had been diagnosed with

bipolar disorder, multiple personality disorder, schizophrenia, autism, intellectual disability, Parkinson's disease; or 2) self-reported bipolar disorder, schizophrenia, or Parkinson's disease; or 3) bipolar disorder indicated by a touchscreen questionnaire assessment (9); or 4) had a prescription for antipsychotic or mood stabilizing medication. Participants were excluded as controls if they 1) had a diagnosis of an anxiety disorder, a mood disorder, or major depressive disorder; or 2) had ever been prescribed antidepressant or anxiolytic medication; 3) self-reported depression; or 4) if they did not provide sufficient data to respond to these questions, or if their responses were intermediate between the control and case definitions described above. In total, 1,198,327 SNPs for 24,015 subjects with putative MDD phenotype available (8,143 cases and 15,872 controls) remained in downstream analyses.

Genotyping, quality control, imputation and phenotyping details in PGC2-MDD dataset

The Psychiatric Genomics Consortium provided lightly quality controlled (Missing-rate < 2%) individual genotypes (best guess) of imputed SNPs for participants from 22 cohorts in PGC2-MDD (Table S1). Subjects who overlapped with GS:SFHS and UK Biobank dataset (108 subjects) were removed. The remained data included 32,554 subjects of European ancestry (13,261 cases and 19,293 controls). We carried out additional QC for the imputed SNPs using the following inclusion thresholds: info score≥0.8, and MAF≥0.01. For each cohort, imputed SNPs that passed QC and could be mapped to the HapMap3 reference panel were used in downstream analyses (Table S1). All cases met DSM-IV criteria for lifetime MDD; the majority of them were ascertained clinically. Most control samples were screened and participants with lifetime MDD were removed (Table S1). Consistent with earlier work (10,11), we grouped the 22 cohorts into 7 groups based on the country of ancestor information for regional heritability analysis (Table S1).

Genome-wide haplotype-block-based regional heritability mapping (HRHM)

To perform HRHM, estimated genome-wide recombination rates expressed as cM/Mb were downloaded from HapMap (phase II) (ftp://ftp.ncbi.nlm.nih.gov/hapmap). For each chromosome, we started from the first base position and expanded the region until it reached a recombination hot-spot where the estimated recombination rate was greater than 10 cM/Mb. This hot-spot marked the end of the region and the start of the next. This process continued until the end of chromosome was reached. Each region was considered to be a haplotype

block. The genotyped SNPs were mapped to 49,637 haplotype-blocks across the genome and the regional heritability was estimated and tested for each of the haplotype-blocks. To test the regional heritability, it is necessary to appropriately account for the polygenic component and the pedigree structure in the analysis model. A standard model incorporates two genomic relationship matrices (GRM); a regional genomic relationship matrix (rGRM) estimated from SNPs in the haplotype block and a complement genomic relationship matrix (cGRM) estimated from all SNPs that are not included in in the haplotype block. These GRMs were jointly fitted in LMM:

$$\boldsymbol{Y} = \boldsymbol{Xb} + \boldsymbol{g_R} + \boldsymbol{g_C} + \boldsymbol{e}$$

$$\mathrm{Var}(\boldsymbol{Y_{random\_effect}}) = \boldsymbol{A_R \sigma_R^2} + \boldsymbol{A_C \sigma_C^2} + \boldsymbol{I \sigma_e^2}$$

$$\boldsymbol{h_R^2} = \boldsymbol{\sigma_R^2 / \sigma_{Y_{random\_effect}}^2}$$

Where $\boldsymbol{Y}$ is a vector of MDD binary phenotypes, and $\boldsymbol{b}$ is a vector of covariates fitted as fixed effects (i.e., age, age^2, sex, 20 principal components derived from the GRM created using all of the genotyped SNPs (fGRM)). $\boldsymbol{g_R}$ and $\boldsymbol{g_C}$ are the random genetic effects from the regional SNPs (in this study, the SNPs that were mapped to the haplotype-block) and the complement set of SNPs, respectively. $\boldsymbol{A_R}$ and $\boldsymbol{A_C}$ are the GRMs created from the regional SNPs and the complement set of SNPs, respectively. The variance explained by the rGRM variance component (regional heritability $\boldsymbol{h_R^2}$) is estimated using restricted estimated maximum likelihood (REML). The estimate is transformed from the observed scale to the liability scale assuming MDD prevalence of 0.13 (5). A log likelihood ratio test (LRT) is applied to test the significance of random effect represented in rGRM by comparing a model with both cGRM and an rGRM fitted against a model including the cGRM but without an rGRM fitted.

The two-GRM model, while providing an unbiased estimate of regional heritability, was highly computationally demanding. To improve the calculation efficiency, a pre-adjustment strategy was applied in the genome-wide HRHM. In detail, we created a genomic relationship matrix using all of the genotyped SNPs (fGRM) (12) and pre-adjusted the random effect represented in the fGRM for the MDD phenotype using the ‘polygenic’ function in GenABEL (13). Residuals (pgresidualY option) were extracted from the model and

used as the phenotype in the HRHM (13). The HRHM was performed by REACTA (14) using the following formula:

$$\boldsymbol{Y}_{gresidual} = \boldsymbol{Xb} + \boldsymbol{g}_{R} + \boldsymbol{e}$$

$$\mathrm{Var}(\boldsymbol{Y}_{gresidual\_random\_effect}) = \boldsymbol{A}_{R}\boldsymbol{\sigma}_{R}^{2} + \boldsymbol{I}\boldsymbol{\sigma}_{e}^{2}$$

$$\boldsymbol{h}_{R}^{2} = \boldsymbol{\sigma}_{R}^{2}/\boldsymbol{\sigma}_{Y\ gresidual\_random\_effect}^{2}$$

Where $\boldsymbol{Y}_{gresidual}$ is a vector of the residuals with fGRM being pre-adjusted, other parameters are the same as in the two-GRM-model. Multiple-testing correction was performed for LRT statistics using the Bonferroni method and the genome-wide significance threshold for HRHM was determined by the number of tests conducted (N_{blocks}=49,637) (15). The genome-wide significance threshold for P values from LRT is 1.01×10^{-6}. For haplotype-blocks that exceeded the genome-wide significant threshold, we re-tested the block using the two-GRM model to provide an accurate estimation of regional heritability in the target block. All the analyses were performed in REACTA (14,16).

Single-haplotype-based association test

Full dataset containing both relatives and non-relatives: Association tests were performed using GCTA-MLMA (12). For each participant, individual haplotypes were coded as 0, 1 or 2. In the linear mixed model, the effect from each haplotype on MDD phenotype was tested one at a time as a fixed effect; other covariates included age, age^2, sex and 20 PCs. As in the single-SNP-based association test, two GRMs were fitted simultaneously as random effects in the model. Bonferroni multiple-testing correction was performed for the P values of each haplotype.

Unrelated dataset (N_{case}=997, $N_{control}$=6367): The dataset was generated by removing one of each pair of individuals with estimated relatedness larger than 0.025 by the function '--grm-cutoff 0.025' in GCTA (12). Association tests on the unrelated dataset were performed using GCTA-MLMA. Since the unrelated dataset contained no pair of individuals with a relatedness larger then 0.025, only the cGRM was fitted in the model.

Case-parent trios ($N_{case\text{-}parents\ trios}$=315): Family-triad based logistic Bayesian Lasso (famLBL) is a method designed for testing the effects of haplotypes on diseases using SNP data using the triad family where the child

is affected by the disease and both the child and parents have genotype and disease diagnosis information available (17). This method shrinks the coefficients of unassociated haplotypes and weighted toward rare haplotypes while retaining sufficient power for detecting common haplotype, allowing for more precise estimation of associated haplotypes (17). The confidence interval (CI) of OR and the Bayes Factor (BF) were given in the results and the BF threshold of 2 was applied (type I error rate ≤ 5%) (17).

Functional annotation tools and analyses for MDD-associated SNPs

(1) Regulomedb is a database providing annotations of known or predicted regulatory functions of non-coding variants. A score was provided for each SNP based on evidence from high-throughput experimental data as well as computational predictions and manual annotations to represent whether the SNP is likely to alter the binding of transcription factors (18).

(2) Genome Wide Annotation of VAriants (GWAVA) is a tool providing prediction of the functional influence of non-coding variants based on annotations of non-coding elements along with genome-wide properties, such as evolutionary conservation and GC-content (19).

(3) Genomic Evolutionary Rate Profiling (GERP) is a method providing estimates of evolutionary constraint with single variant resolution using maximum likelihood evolutionary rate estimation. A score threshold of 2 provides high sensitivity while still strongly enriching for truly constrained sites (20).

(4) Allelic effect on gene-expression (eQTL analysis). In GTEX (http://www.gtexportal.org/home/datasets), tissue-specific SNP-*cis*-gene association test results were available from 11 brain tissues (a *cis* window was defined as +/- 1MB around the transcript start site (TSS)). The information available includes test statistics (a two tailed t-test) of the effect of the alternative allele relative to the reference allele on the expression of the gene for each SNP-gene pair in each tissue. We downloaded the 11 files and only extracted the records for the 38 SNPs. In BRAINEAC, tissue-specific SNP-*cis*-gene association test results were available from 10 brain tissues. P values from the tissue-specific *cis*-eQTL analyses were downloaded from http://caprica.genetics.kcl.ac.uk/BRAINEAC/. In results extracted from both GTEX and BRAINEAC, FDR multiple testing correction was applied to each tissue separately using the R package ‘qvalue’ (21) ($N_{correction}=N_{cis\text{-}genes}*N_{snps}$).

(5) Allelic effect on DNA methylation in CpG loci (meQTL analysis). Jaffe *et al.* (2016) identified meQTLs in frontal cortex using SNP-cis-CpG DNA methylation analysis on 258 adult control samples (age > 13) (22). Additionally, they also identified CpG loci that have differential DNA methylation level between fetal and postnatal life, which suggests a role of those loci in the early development stage in frontal cortex. We extracted the records of the 38 SNPs from the downloaded summary statistics file of the SNP-cis-CpG DNA methylation analysis; this included FDR values for each pair of associations and the estimate of the SNP effect (Beta). For the CpG loci that were significantly regulated by the 38 SNPs, we further checked the summary statistics file of the CpG loci differentially DNA methylated between fetal and postnatal life to see whether the regulated CpG is likely to play a role in the development of frontal cortex.

Table S1. Individual cohort and grouping information for 22 cohorts in PGC2-MDD. According to the country of ascertainment , the 22 cohorts were grouped into seven groups for the replication analysis.

Group	Country of Ascertainment	Cohort	N_snps	N_cases	N_controls	Study Name	Case Definition	Screened Control
AUS	Australia	cof3	779887	120	126	COFAMS	MDD	yes
AUS	Australia	qi3c	832512	863	579	QIMR I317	MDD	yes
AUS	Australia	qi6c	1012990	499	590	QIMR I610	MDD	yes
AUS	Australia	qio2	781801	565	526	QIMR COEX	MDD	yes
GER	Germany	boma	1028670	586	1062	BOMA	MDD	no
GER	Germany	gsk2	1035814	879	860	GSK MPIP	rMDD	yes
GER	Germany	mmi2	828603	584	517	MPIP MARS 650	MDD	yes
GER	Germany	mmo4	1053857	264	371	MPIP MARS OMNIex	MDD	yes
GER	Germany	rage	1009799	322	227	RADIANT - German cases	rMDD	no
MIXED	Switzerland	col3	721251	506	1445	PsyCoLaus	MDD	yes
MIXED	Denmark	rde4	847865	133	516	RADIANT - Danish cases	rMDD	yes
NET	Netherlands	nes1	932158	1494	1602	NTR/NESDA	MDD	yes
NET	Netherlands	rot4	1029761	241	1028	Rotterdam	MDD	yes
SWE	Sweden	twg2	1089388	1097	2663	TwinGene	MDD	yes
UK_IRE	Scotland	edi2	950236	363	283	Edinburgh	rMDD	yes
UK_IRE	UK	gep3	1031973	472	2814	GENPOD/NewMeds	MDD	no
UK_IRE	UK	rad3	1040419	1835	1357	RADIANT	rMDD	yes
UK_IRE	Ireland	rai2	983451	109	339	RADIANT - Irish cases	rMDD	yes
USA	USA	grdg	1058400	471	470	DepGenesNetwork	MDD	yes
USA	USA	grnd	1063155	829	474	GenRED2	reoMDD	yes
USA	USA	i2b3	1034048	806	1066	Harvard i2b2	MDD	yes
USA	USA	rau2	1011577	223	378	RADIANT - US cases	rMDD	no

Table S2. Single-SNP-based association test results for nine genotyped SNPs in the hit haplotype block in GS:SFHS. Beta(linear): estimates on the linear scale, OR was obtained using taylor series approximation. Adjusted P: Bonferroni method adjusted P value. *: significant results.

rs ID	Chr	Pos	A1	A2	Freq	Beta (linear)	SE (Beta (linear))	OR	logOR	SE (logOR)	P	Adjusted P
**rs6017218**	20	42555737	G(C)	T(A)	0.126	-0.019	0.005	0.833	-0.183	0.041	2.44E-04	2.19E-03 *
**rs6031242**	20	42556096	G(C)	A(T)	0.113	-0.019	0.006	0.832	-0.184	0.043	4.36E-04	3.93E-03 *
**rs17753711**	20	42559319	A(T)	G(C)	0.305	-0.002	0.004	0.981	-0.019	0.033	5.76E-01	1.00E+00
**rs6031245**	20	42559531	T(A)	C(G)	0.096	-0.025	0.006	0.783	-0.244	0.045	2.30E-05	2.07E-04 *
**rs6031247**	20	42563647	A(T)	G(C)	0.147	-0.010	0.005	0.915	-0.089	0.041	4.89E-02	4.40E-01
**rs6093898**	20	42566577	G(C)	A(T)	0.097	-0.025	0.006	0.783	-0.245	0.045	2.03E-05	1.83E-04 *
**rs4812767**	20	42568829	T(A)	C(G)	0.096	-0.025	0.006	0.785	-0.242	0.045	2.57E-05	2.31E-04 *
**rs11700304**	20	42574362	C(G)	T(A)	0.374	-0.004	0.004	0.968	-0.032	0.031	3.17E-01	1.00E+00
**rs4812772**	20	42579051	T(A)	C(G)	0.261	0.008	0.004	1.072	0.070	0.037	4.19E-02	3.77E-01

Table S3. Haplotype-based association test results for common haplotypes derived from nine common SNPs in hit block on unrelated individual dataset of GS:SFHS. *: significant result.

Haplotype	Freq	Beta (linear)	SE (Beta (linear))	OR	logOR	SE (logOR)	P	Adjusted P
**TAGCGACCT**	0.119	0.030	0.009	1.275	0.243	0.101	4.82E-04	3.85E-03 *
**GGGCGACCT**	0.010	0.047	0.028	1.440	0.364	0.371	9.26E-02	7.41E-01
**GGGTGGTCC**	0.094	-0.011	0.010	0.902	-0.103	0.079	2.47E-01	1.00E+00
**TAGCGACTC**	0.310	-0.005	0.006	0.957	-0.043	0.052	4.25E-01	1.00E+00
**GAGCAACCT**	0.012	-0.020	0.025	0.827	-0.190	0.196	4.26E-01	1.00E+00
**TAGCAACCT**	0.118	-0.007	0.009	0.942	-0.059	0.073	4.44E-01	1.00E+00
**TAGCAACCC**	0.015	-0.013	0.023	0.888	-0.119	0.188	5.79E-01	1.00E+00
**TATCGACTC**	0.307	-0.001	0.006	0.993	-0.007	0.053	9.00E-01	1.00E+00

Table S4. Haplotype-based association test results for common haplotypes derived from nine common SNPs in hit block on case-parents-trio of GS:SFHS. The BF threshold of 2 was applied for significance (type I error rate ≤5%). *: significant result.

Haplotype	Frequency	OR	Lower OR	Upper OR	BF
**GGGTGGTCC**	0.119	0.573	0.376	0.858	8.113 *
**GAGCAACCT**	0.014	0.577	0.191	1.348	0.806
**GGGCGACTC**	0.013	0.733	0.268	1.648	0.473
**TAGCAACCC**	0.018	1.083	0.559	2.185	0.282
**TAGCAACCT**	0.109	0.892	0.617	1.245	0.170
**TAGCGACCT**	0.120	1.126	0.824	1.569	0.167
**TATCGACTC**	0.285	0.888	0.687	1.151	0.155

Table S5. Results of single-SNP-based association test on 53 imputed common SNPs in the hit block in GS:SFHS. Adjusted P: Bonferroni method adjusted P value. *: significant results.

rs id	Chr	Pos	A1	A2	Freq	Beta (linear)	SE (Beta (linear))	OR	logOR	SE (logOR)	P	Adjusted P
**rs4812766**	20	42559149	A	G	0.096	-0.026	0.006	0.780	-0.248	0.045	1.72E-05	9.10E-04 *
**rs11905261**	20	42566120	C	A	0.096	-0.025	0.006	0.782	-0.246	0.045	1.99E-05	1.05E-03 *
**rs78295570**	20	42567815	T	C	0.097	-0.025	0.006	0.783	-0.245	0.045	2.00E-05	1.06E-03 *
**rs6093898**	20	42566577	G	A	0.097	-0.025	0.006	0.783	-0.245	0.045	2.03E-05	1.08E-03 *
**rs6103524**	20	42568152	G	A	0.097	-0.025	0.006	0.783	-0.245	0.045	2.03E-05	1.08E-03 *
**rs77591323**	20	42567817	C	T	0.097	-0.025	0.006	0.783	-0.245	0.045	2.10E-05	1.11E-03 *
**rs6031245**	20	42559531	T	C	0.096	-0.025	0.006	0.783	-0.244	0.045	2.30E-05	1.22E-03 *
**rs75074505**	20	42567941	G	C	0.097	-0.025	0.006	0.784	-0.243	0.045	2.31E-05	1.22E-03 *
**rs1888982**	20	42566009	A	G	0.097	-0.025	0.006	0.784	-0.243	0.045	2.31E-05	1.23E-03 *
**rs916474**	20	42563571	A	G	0.096	-0.025	0.006	0.784	-0.244	0.045	2.36E-05	1.25E-03 *
**rs79225010**	20	42557504	A	G	0.096	-0.025	0.006	0.784	-0.244	0.045	2.38E-05	1.26E-03 *
**rs868981**	20	42562557	A	G	0.096	-0.025	0.006	0.784	-0.243	0.045	2.46E-05	1.30E-03 *
**rs11086910**	20	42579473	A	G	0.095	-0.025	0.006	0.783	-0.244	0.045	2.49E-05	1.32E-03 *
**rs4812767**	20	42568829	T	C	0.096	-0.025	0.006	0.785	-0.242	0.045	2.57E-05	1.36E-03 *
**rs944703**	20	42564176	C	A	0.096	-0.025	0.006	0.785	-0.242	0.045	2.62E-05	1.39E-03 *
**rs79645278**	20	42568164	T	C	0.096	-0.025	0.006	0.785	-0.242	0.045	2.67E-05	1.41E-03 *
**rs2002343**	20	42570003	A	G	0.096	-0.025	0.006	0.785	-0.242	0.045	2.67E-05	1.41E-03 *
**rs4383390**	20	42572303	C	T	0.096	-0.025	0.006	0.785	-0.242	0.045	2.67E-05	1.41E-03 *
**rs56852079**	20	42562168	A	G	0.096	-0.025	0.006	0.786	-0.241	0.045	2.85E-05	1.51E-03 *
**rs57150899**	20	42562169	T	C	0.096	-0.025	0.006	0.786	-0.241	0.045	2.86E-05	1.52E-03 *
**rs4812770**	20	42575630	G	C	0.097	-0.025	0.006	0.789	-0.237	0.045	3.62E-05	1.92E-03 *
**rs11907990**	20	42556041	T	C	0.096	-0.024	0.006	0.790	-0.235	0.045	4.24E-05	2.24E-03 *
**rs11906108**	20	42556057	T	A	0.096	-0.024	0.006	0.790	-0.235	0.045	4.24E-05	2.24E-03 *
**rs76623859**	20	42555756	A	C	0.096	-0.024	0.006	0.791	-0.235	0.045	4.47E-05	2.37E-03 *
**rs910909**	20	42575799	G	A	0.096	-0.024	0.006	0.792	-0.233	0.045	4.84E-05	2.57E-03 *
**rs910907**	20	42576998	T	G	0.095	-0.024	0.006	0.793	-0.231	0.045	5.85E-05	3.10E-03 *
**rs4812771**	20	42575733	T	C	0.095	-0.024	0.006	0.793	-0.231	0.045	5.87E-05	3.11E-03 *
**rs910908**	20	42575841	A	G	0.095	-0.024	0.006	0.793	-0.231	0.045	5.87E-05	3.11E-03 *
**rs6093895**	20	42557501	A	G	0.118	-0.021	0.005	0.819	-0.200	0.042	1.08E-04	5.71E-03 *
**rs6130482**	20	42557999	C	T	0.118	-0.021	0.005	0.819	-0.200	0.042	1.09E-04	5.77E-03 *
**rs743152**	20	42569911	T	G	0.106	-0.022	0.006	0.811	-0.209	0.044	1.20E-04	6.34E-03 *
**rs6017223**	20	42571020	G	C	0.106	-0.022	0.006	0.814	-0.206	0.044	1.60E-04	8.47E-03 *
**rs6017220**	20	42564336	C	T	0.107	-0.021	0.006	0.816	-0.204	0.044	1.72E-04	9.12E-03 *
**rs6103521**	20	42566398	G	T	0.106	-0.021	0.006	0.816	-0.203	0.044	1.75E-04	9.28E-03 *
**rs6103517**	20	42558608	A	G	0.118	-0.020	0.005	0.825	-0.192	0.042	1.92E-04	1.02E-02 *
**rs6017218**	20	42555737	G	T	0.126	-0.019	0.005	0.833	-0.183	0.041	2.44E-04	1.29E-02 *
**rs6031242**	20	42556096	G	A	0.113	-0.019	0.006	0.832	-0.184	0.043	4.36E-04	2.31E-02 *
**rs6103516**	20	42556151	G	A	0.113	-0.019	0.006	0.834	-0.181	0.043	5.39E-04	2.86E-02 *
**rs138949844**	20	42570224	T	C	0.026	0.027	0.011	1.245	0.219	0.120	1.51E-02	1.00E+00
**rs4812773**	20	42579148	T	C	0.261	0.008	0.004	1.074	0.071	0.037	3.79E-02	1.00E+00
**rs4812772**	20	42579051	T	C	0.261	0.008	0.004	1.072	0.070	0.037	4.19E-02	1.00E+00

rs id	Chr	Pos	A1	A2	Freq	Beta (linear)	SE (Beta (linear))	OR	logOR	SE (logOR)	P	Adjusted P
**rs6031247**	20	42563647	A	G	0.147	-0.010	0.005	0.915	-0.089	0.041	4.89E-02	1.00E+00
**rs141048930**	20	42567378	G	T	0.015	-0.027	0.014	0.767	-0.266	0.106	5.59E-02	1.00E+00
**rs4812769**	20	42574442	T	C	0.251	0.007	0.004	1.065	0.063	0.038	6.94E-02	1.00E+00
**rs73118242**	20	42568478	C	T	0.012	-0.017	0.016	0.853	-0.159	0.128	2.94E-01	1.00E+00
**rs11700304**	20	42574362	C	T	0.374	-0.004	0.004	0.968	-0.032	0.031	3.17E-01	1.00E+00
**rs4812774**	20	42579188	C	T	0.373	-0.004	0.004	0.969	-0.031	0.031	3.33E-01	1.00E+00
**rs76571595**	20	42572032	G	T	0.012	-0.014	0.016	0.879	-0.129	0.129	3.82E-01	1.00E+00
**rs6031252**	20	42573822	A	C	0.010	0.015	0.018	1.137	0.128	0.174	3.89E-01	1.00E+00
**rs761919**	20	42577362	C	T	0.372	-0.003	0.004	0.973	-0.028	0.031	3.93E-01	1.00E+00
**rs73118230**	20	42556503	C	T	0.012	-0.011	0.016	0.908	-0.097	0.131	5.06E-01	1.00E+00
**rs79663350**	20	42573034	A	G	0.028	0.001	0.011	1.009	0.009	0.095	9.20E-01	1.00E+00
**rs8117150**	20	42569690	A	G	0.015	0.001	0.015	1.007	0.007	0.130	9.59E-01	1.00E+00

Table S6. The functional prediction of 38 significant SNPs using regulomeDB score, GWAVA-Tss score and GERP score.

rs id	Pos	Chr	A1	RegulomeDB	GWAVA-TSS	GERP
**rs6017218**	42555737	20	G	4	0.180	3.340
**rs76623859**	42555756	20	A	2b	0.180	-0.046
**rs11907990**	42556041	20	T	4	0.290	-2.900
**rs11906108**	42556057	20	T	4	0.240	-3.340
**rs6031242**	42556096	20	G	5	0.350	-5.020
**rs6103516**	42556151	20	G	5	0.120	-0.218
**rs6093895**	42557501	20	A	5	0.150	-1.560
**rs79225010**	42557504	20	A	5	0.130	0.780
**rs6130482**	42557999	20	C	5	0.090	-3.660
**rs6103517**	42558608	20	A	7	0.090	-0.633
**rs4812766**	42559149	20	A	5	0.180	1.860
**rs6031245**	42559531	20	T	7	0.360	-3.070
**rs56852079**	42562168	20	A	5	0.330	-4.140
**rs57150899**	42562169	20	T	5	0.190	-1.260
**rs868981**	42562557	20	A	5	0.330	2.250
**rs916474**	42563571	20	A	5	0.310	0.489
**rs944703**	42564176	20	C	5	0.180	-4.940
**rs6017220**	42564336	20	C	6	0.160	0.235
**rs1888982**	42566009	20	A	5	0.280	-2.420
**rs11905261**	42566120	20	C	5	0.300	0.964
**rs6103521**	42566398	20	G	4	0.240	-3.570
**rs6093898**	42566577	20	G	4	0.200	0.442
**rs78295570**	42567815	20	T	4	0.470	-5.270
**rs77591323**	42567817	20	C	4	0.530	-5.770
**rs75074505**	42567941	20	G	4	0.400	-1.570
**rs6103524**	42568152	20	G	4	0.510	2.290
**rs79645278**	**42568164**	**20**	**T**	**2b**	**0.500**	**2.310**
**rs4812767**	42568829	20	T	4	0.350	-0.751
**rs743152**	42569911	20	T	6	0.380	-1.980
**rs2002343**	42570003	20	A	7	0.330	-0.470
**rs6017223**	42571020	20	G	3a	0.510	-4.170
**rs4383390**	42572303	20	C	6	0.520	-2.190
**rs4812770**	42575630	20	G	5	0.120	1.370
**rs4812771**	42575733	20	T	7	0.180	2.090
**rs910909**	42575799	20	G	7	0.130	1.310
**rs910908**	42575841	20	A	6	0.120	-2.430
**rs910907**	42576998	20	T	5	0.200	-1.160
**rs11086910**	42579473	20	A	4	0.300	-1.820

Table S7. Significant results (Q value ≤ 0.05) from SNP-cis-gene-eQTL analysis using GTEX. The analysis was performed on 11 brain tissues in GTEX and significant results were obtained from the tissue 'Nucleus accumbens basal ganglia'. A1 is the tested allele, the same allele tested in single-SNP-based association test. Q value: Adjusted P values (FDR).

rs id	Pos	Chr	A1	Gene name	Gene_pos	eQTL_beta	eQTL_P	eQTL_Q value
**rs78295570**	42567815	20	T	RP1-269M15.3	20_41818862-41830011	0.373132	2.35E-04	3.17E-02
**rs77591323**	42567817	20	C	RP1-269M15.3	20_41818862-41830011	0.373132	2.35E-04	3.17E-02
**rs75074505**	42567941	20	G	RP1-269M15.3	20_41818862-41830011	unsigned_0.373132	2.35E-04	3.17E-02
**rs6103524**	42568152	20	G	RP1-269M15.3	20_41818862-41830011	0.373132	2.35E-04	3.17E-02
**rs1888982**	42566009	20	A	RP1-269M15.3	20_41818862-41830011	0.373229	2.36E-04	3.17E-02
**rs56852079**	42562168	20	A	RP1-269M15.3	20_41818862-41830011	0.357319	2.52E-04	3.17E-02
**rs910909**	42575799	20	G	RP1-269M15.3	20_41818862-41830011	0.352567	2.94E-04	3.17E-02
**rs11086910**	42579473	20	A	RP1-269M15.3	20_41818862-41830011	0.381632	4.45E-04	4.10E-02
**rs6093898**	42566577	20	G	RP1-269M15.3	20_41818862-41830011	0.357227	4.89E-04	4.10E-02
**rs743152**	42569911	20	T	RP1-269M15.3	20_41818862-41830011	0.3497	8.18E-04	4.25E-02
**rs6103521**	42566398	20	G	RP1-269M15.3	20_41818862-41830011	0.336035	8.66E-04	4.25E-02
**rs6017220**	42564336	20	C	RP1-269M15.3	20_41818862-41830011	0.335674	8.85E-04	4.25E-02
**rs11905261**	42566120	20	C	RP1-269M15.3	20_41818862-41830011	0.338646	9.06E-04	4.25E-02
**rs11906108**	42556057	20	T	RP1-269M15.3	20_41818862-41830011	unsigned_0.35263	1.09E-03	4.25E-02
**rs868981**	42562557	20	A	RP1-269M15.3	20_41818862-41830011	0.31452	1.18E-03	4.25E-02
**rs57150899**	42562169	20	T	RP1-269M15.3	20_41818862-41830011	0.361794	1.41E-03	4.25E-02
**rs11907990**	42556041	20	T	RP1-269M15.3	20_41818862-41830011	0.347766	1.46E-03	4.25E-02
**rs4383390**	42572303	20	C	RP1-269M15.3	20_41818862-41830011	0.345133	1.59E-03	4.25E-02
**rs6031245**	42559531	20	T	RP1-269M15.3	20_41818862-41830011	0.345064	1.59E-03	4.25E-02
**rs79645278**	42568164	20	T	RP1-269M15.3	20_41818862-41830011	0.345064	1.59E-03	4.25E-02
**rs4812767**	42568829	20	T	RP1-269M15.3	20_41818862-41830011	0.345064	1.59E-03	4.25E-02
**rs2002343**	42570003	20	A	RP1-269M15.3	20_41818862-41830011	0.345064	1.59E-03	4.25E-02
**rs4812771**	42575733	20	T	RP1-269M15.3	20_41818862-41830011	0.345064	1.59E-03	4.25E-02
**rs910908**	42575841	20	A	RP1-269M15.3	20_41818862-41830011	0.345064	1.59E-03	4.25E-02
**rs910907**	42576998	20	T	RP1-269M15.3	20_41818862-41830011	0.345064	1.59E-03	4.25E-02
**rs4812766**	42559149	20	A	RP1-269M15.3	20_41818862-41830011	0.344928	1.60E-03	4.25E-02
**rs79225010**	42557504	20	A	RP1-269M15.3	20_41818862-41830011	0.344801	1.60E-03	4.25E-02
**rs944703**	42564176	20	C	RP1-269M15.3	20_41818862-41830011	0.344844	1.62E-03	4.25E-02
**rs916474**	42563571	20	A	RP1-269M15.3	20_41818862-41830011	0.344701	1.63E-03	4.25E-02
**rs76623859**	42555756	20	A	RP1-269M15.3	20_41818862-41830011	0.344566	1.71E-03	4.29E-02

Table S8. Significant results (Q value ≤ 0.05) from SNP-cis-gene-eQTL analysis on frontal cortex using BRAINEAC. The analysis was performed on 10 brain tissues in BRAINEAC and significant results were obtained from two tissues: frontal cortex and cerebellar cortex. A1 is the tested allele, the same allele tested in single-SNP-based association test. P: p values from SNP-cis-gene-eQTL analysis. Q: Adjusted P values (FDR).

Gene Symbol	rs id	Chr	Pos	exprID	Gene_start	Gene_end	P	Q
**TOX2,LOC100128170**	rs4383390	20	42572303	t3886294	42543004	42710556	1.80E-05	3.06E-04
**TOX2,LOC100128170**	rs2002343	20	42570003	t3886294	42543004	42710556	1.80E-05	3.06E-04
**TOX2,LOC100128170**	rs868981	20	42562557	t3886294	42543004	42710556	1.80E-05	3.06E-04
**TOX2,LOC100128170**	rs916474	20	42563571	t3886294	42543004	42710556	1.80E-05	3.06E-04
**TOX2,LOC100128170**	rs944703	20	42564176	t3886294	42543004	42710556	1.80E-05	3.06E-04
**TOX2,LOC100128170**	rs79225010	20	42557504	t3886294	42543004	42710556	1.80E-05	3.06E-04
**TOX2,LOC100128170**	rs6031245	20	42559531	t3886294	42543004	42710556	1.80E-05	3.06E-04
**TOX2,LOC100128170**	rs6093898	20	42566577	t3886294	42543004	42710556	1.80E-05	3.06E-04
**TOX2,LOC100128170**	rs78295570	20	42567815	t3886294	42543004	42710556	1.80E-05	3.06E-04
**TOX2,LOC100128170**	rs77591323	20	42567817	t3886294	42543004	42710556	1.80E-05	3.06E-04
**TOX2,LOC100128170**	rs75074505	20	42567941	t3886294	42543004	42710556	1.80E-05	3.06E-04
**TOX2,LOC100128170**	rs6103524	20	42568152	t3886294	42543004	42710556	1.80E-05	3.06E-04
**TOX2,LOC100128170**	rs79645278	20	42568164	t3886294	42543004	42710556	1.80E-05	3.06E-04
**TOX2,LOC100128170**	rs4812767	20	42568829	t3886294	42543004	42710556	1.80E-05	3.06E-04
**TOX2,LOC100128170**	rs4812766	20	42559149	t3886294	42543004	42710556	1.80E-05	3.06E-04
**TOX2,LOC100128170**	rs1888982	20	42566009	t3886294	42543004	42710556	1.80E-05	3.06E-04
**TOX2,LOC100128170**	rs11905261	20	42566120	t3886294	42543004	42710556	1.80E-05	3.06E-04
**TOX2,LOC100128170**	rs910909	20	42575799	t3886294	42543004	42710556	1.80E-05	3.06E-04
**TOX2,LOC100128170**	rs910908	20	42575841	t3886294	42543004	42710556	1.80E-05	3.06E-04
**TOX2,LOC100128170**	rs4812771	20	42575733	t3886294	42543004	42710556	1.80E-05	3.06E-04
**TOX2,LOC100128170**	rs76623859	20	42555756	t3886294	42543004	42710556	1.80E-05	3.06E-04
**TOX2,LOC100128170**	rs11907990	20	42556041	t3886294	42543004	42710556	1.80E-05	3.06E-04
**TOX2,LOC100128170**	rs11906108	20	42556057	t3886294	42543004	42710556	1.80E-05	3.06E-04
**TOX2,LOC100128170**	rs910907	20	42576998	t3886294	42543004	42710556	1.80E-05	3.06E-04
**TOX2,LOC100128170**	rs743152	20	42569911	t3886294	42543004	42710556	1.60E-05	3.06E-04
**TOX2,LOC100128170**	rs6017223	20	42571020	t3886294	42543004	42710556	1.60E-05	3.06E-04
**TOX2,LOC100128170**	rs6103521	20	42566398	t3886294	42543004	42710556	1.60E-05	3.06E-04
**TOX2,LOC100128170**	rs6017220	20	42564336	t3886294	42543004	42710556	1.60E-05	3.06E-04
**TOX2,LOC100128170**	rs11086910	20	42579473	t3886294	42543004	42710556	1.80E-05	3.06E-04
**TOX2,LOC100128170**	rs6031242	20	42556096	t3886294	42543004	42710556	5.00E-06	3.06E-04
**TOX2,LOC100128170**	rs6103516	20	42556151	t3886294	42543004	42710556	5.00E-06	3.06E-04
**TOX2,LOC100128170**	rs4812770	20	42575630	t3886294	42543004	42710556	2.00E-05	3.29E-04
**TOX2,LOC100128170**	rs6017218	20	42555737	t3886294	42543004	42710556	2.10E-05	3.35E-04
**TOX2,LOC100128170**	rs6130482	20	42557999	t3886294	42543004	42710556	6.70E-05	1.04E-03
**TOX2,LOC100128170**	rs6093895	20	42557501	t3886294	42543004	42710556	6.90E-05	1.04E-03
**TOX2,LOC100128170**	rs57150899	20	42562169	t3886294	42543004	42710556	0.00026	3.70E-03
**TOX2,LOC100128170**	rs56852079	20	42562168	t3886294	42543004	42710556	0.00026	3.70E-03
**TOX2,LOC100128170**	rs6103517	20	42558608	t3886294	42543004	42710556	0.00078	1.08E-02

Table S9. Significant results (Q value ≤ 0.05) from SNP-cis-gene-eQTL analysis on cerebellar cortex using BRAINEAC. The analysis was performed on 10 brain tissues in BRAINEAC and significant results were obtained from two tissues: frontal cortex and cerebellar cortex. A1 is the tested allele, the same allele tested in single-SNP-based association test. P: p values from SNP-cis-gene-eQTL analysis. Q: Adjusted P values (FDR).

Gene Symbol	Pos	Chr	rs id	exprID	chr	Gene_start	Gene_end	P	Q
**C20orf62**	42572303	20	rs4383390	t3906923	chr20	43080624	43093984	8.70E-04	2.34E-02
**C20orf62**	42566009	20	rs1888982	t3906923	chr20	43080624	43093984	8.70E-04	2.34E-02
**C20orf62**	42566120	20	rs11905261	t3906923	chr20	43080624	43093984	8.70E-04	2.34E-02
**C20orf62**	42570003	20	rs2002343	t3906923	chr20	43080624	43093984	8.80E-04	2.34E-02
**C20orf62**	42563571	20	rs916474	t3906923	chr20	43080624	43093984	8.80E-04	2.34E-02
**C20orf62**	42564176	20	rs944703	t3906923	chr20	43080624	43093984	8.80E-04	2.34E-02
**C20orf62**	42559531	20	rs6031245	t3906923	chr20	43080624	43093984	8.80E-04	2.34E-02
**C20orf62**	42566577	20	rs6093898	t3906923	chr20	43080624	43093984	8.80E-04	2.34E-02
**C20orf62**	42567815	20	rs78295570	t3906923	chr20	43080624	43093984	8.80E-04	2.34E-02
**C20orf62**	42567817	20	rs77591323	t3906923	chr20	43080624	43093984	8.80E-04	2.34E-02
**C20orf62**	42567941	20	rs75074505	t3906923	chr20	43080624	43093984	8.80E-04	2.34E-02
**C20orf62**	42568152	20	rs6103524	t3906923	chr20	43080624	43093984	8.80E-04	2.34E-02
**C20orf62**	42568164	20	rs79645278	t3906923	chr20	43080624	43093984	8.80E-04	2.34E-02
**C20orf62**	42568829	20	rs4812767	t3906923	chr20	43080624	43093984	8.80E-04	2.34E-02
**C20orf62**	42562557	20	rs868981	t3906923	chr20	43080624	43093984	8.80E-04	2.34E-02
**C20orf62**	42559149	20	rs4812766	t3906923	chr20	43080624	43093984	8.80E-04	2.34E-02
**C20orf62**	42575630	20	rs4812770	t3906923	chr20	43080624	43093984	8.50E-04	2.34E-02
**C20orf62**	42575841	20	rs910908	t3906923	chr20	43080624	43093984	8.70E-04	2.34E-02
**C20orf62**	42575733	20	rs4812771	t3906923	chr20	43080624	43093984	8.70E-04	2.34E-02
**C20orf62**	42575799	20	rs910909	t3906923	chr20	43080624	43093984	8.70E-04	2.34E-02
**C20orf62**	42555756	20	rs76623859	t3906923	chr20	43080624	43093984	8.80E-04	2.34E-02
**C20orf62**	42556041	20	rs11907990	t3906923	chr20	43080624	43093984	8.80E-04	2.34E-02
**C20orf62**	42556057	20	rs11906108	t3906923	chr20	43080624	43093984	8.80E-04	2.34E-02
**C20orf62**	42564336	20	rs6017220	t3906923	chr20	43080624	43093984	8.90E-04	2.34E-02
**C20orf62**	42566398	20	rs6103521	t3906923	chr20	43080624	43093984	8.90E-04	2.34E-02
**C20orf62**	42569911	20	rs743152	t3906923	chr20	43080624	43093984	9.00E-04	2.34E-02
**C20orf62**	42571020	20	rs6017223	t3906923	chr20	43080624	43093984	9.00E-04	2.34E-02
**C20orf62**	42576998	20	rs910907	t3906923	chr20	43080624	43093984	8.70E-04	2.34E-02
**C20orf62**	42557504	20	rs79225010	t3906923	chr20	43080624	43093984	9.10E-04	2.34E-02
**C20orf62**	42579473	20	rs11086910	t3906923	chr20	43080624	43093984	8.70E-04	2.34E-02
**C20orf62**	42562169	20	rs57150899	t3906923	chr20	43080624	43093984	2.80E-04	2.34E-02
**C20orf62**	42562168	20	rs56852079	t3906923	chr20	43080624	43093984	2.80E-04	2.34E-02
**C20orf62**	42556096	20	rs6031242	t3906923	chr20	43080624	43093984	1.10E-03	2.66E-02
**C20orf62**	42556151	20	rs6103516	t3906923	chr20	43080624	43093984	1.10E-03	2.66E-02
**C20orf62**	42558608	20	rs6103517	t3906923	chr20	43080624	43093984	2.70E-03	6.35E-02

Table S10. Significant results of meQTL SNPs for CpG locus cg24403644 (FDR ≤ 0.05) from SNP-cis-CpG DNA methylation analysis on frontal cortex.

SNP Chr	SNP Pos	SNP Rs Num	SNP Counted	CpG	Meth Chr	Meth Pos	beta	P value	FDR
chr20	42563571	rs916474	A	cg24403644	chr20	42574624	-0.02	9.99E-08	2.80E-06
chr20	42564176	rs944703	C	cg24403644	chr20	42574624	-0.02	1.66E-07	4.48E-06
chr20	42566009	rs1888982	A	cg24403644	chr20	42574624	-0.01	2.58E-08	7.94E-07
chr20	42566120	rs11905261	C	cg24403644	chr20	42574624	-0.01	2.31E-07	6.09E-06
chr20	42566577	rs6093898	G	cg24403644	chr20	42574624	-0.01	9.27E-07	2.20E-05
chr20	42567815	rs78295570	T	cg24403644	chr20	42574624	-0.01	2.88E-07	7.47E-06
chr20	42567817	rs77591323	C	cg24403644	chr20	42574624	-0.01	2.88E-07	7.47E-06
chr20	42567941	rs75074505	G	cg24403644	chr20	42574624	-0.01	6.72E-07	1.63E-05
chr20	42568152	rs6103524	G	cg24403644	chr20	42574624	-0.01	2.88E-07	7.47E-06
chr20	42568164	rs79645278	T	cg24403644	chr20	42574624	-0.02	1.00E-07	2.81E-06
chr20	42568829	rs4812767	T	cg24403644	chr20	42574624	-0.02	1.00E-07	2.81E-06
chr20	42570003	rs2002343	A	cg24403644	chr20	42574624	-0.01	1.14E-06	2.65E-05
chr20	42572303	rs4383390	C	cg24403644	chr20	42574624	-0.01	1.14E-06	2.65E-05
chr20	42575630	rs4812770	G	cg24403644	chr20	42574624	-0.02	3.17E-07	8.17E-06
chr20	42575733	rs4812771	T	cg24403644	chr20	42574624	-0.02	2.68E-07	7.00E-06
chr20	42575799	rs910909	G	cg24403644	chr20	42574624	-0.01	6.87E-05	1.09E-03
chr20	42575841	rs910908	A	cg24403644	chr20	42574624	-0.02	3.41E-07	8.75E-06
chr20	42576998	rs910907	T	cg24403644	chr20	42574624	-0.02	9.77E-08	2.75E-06
chr20	42579473	rs11086910	A	cg24403644	chr20	42574624	-0.01	5.12E-08	1.50E-06

Table S11. Regional heritability estimates of the hit block in seven groups and the combined (22 cohorts) in PGC2-MDD and UK Biobank. $\boldsymbol{h_R^2}$: regional heritability. P_{lrt}: P value of LRT for $\boldsymbol{h_R^2}$.

Replication Sample	Group	P_{lrt}	h_R^2	se(h_R^2)	h_C^2	se(h_C^2)
**PGC2-MDD**	Combined 22	0.500	0.000	0.000	0.139	0.012
**PGC2-MDD**	AUS	the matrix V becomes negative-definite				
**PGC2-MDD**	GER	0.415	0.000	0.001	0.357	0.064
**PGC2-MDD**	MIXED	0.500	0.000	0.002	0.157	0.159
**PGC2-MDD**	NET	0.500	0.000	0.001	0.351	0.081
**PGC2-MDD**	SWE	0.068	0.003	0.004	0.341	0.115
**PGC2-MDD**	UK-Ireland	0.049*	0.001	0.001	0.227	0.051
**PGC2-MDD**	USA	0.500	0.000	0.001	0.252	0.079
**UK Biobank**	UK Biobank	0.151	0.000	0.000	0.130	0.019

Table S12. Single-SNP-based association tests for five SNPs in individual cohorts PGC2-MDD.

rs id	Pos	Chr	Cohort	A1	OR	logOR	SE (logOR)	P
**rs6017218**	42555737	20	boma	G	1.006	0.006	0.120	0.960
**rs6017218**	42555737	20	cof3	G	0.746	-0.293	0.279	0.294
**rs6017218**	42555737	20	gep3	G	0.916	-0.087	0.180	0.626
**rs6017218**	42555737	20	grdg	G	0.966	-0.035	0.139	0.801
**rs6017218**	42555737	20	grnd	G	0.939	-0.063	0.131	0.632
**rs6017218**	42555737	20	gsk2	G	0.924	-0.079	0.103	0.446
**rs6017218**	42555737	20	i2b3	G	1.160	0.148	0.100	0.139
**rs6017218**	42555737	20	mmi2	G	1.150	0.140	0.130	0.284
**rs6017218**	42555737	20	mmo4	G	1.089	0.085	0.213	0.690
**rs6017218**	42555737	20	qi3c	G	0.968	-0.032	0.114	0.776
**rs6017218**	42555737	20	qi6c	G	1.019	0.019	0.131	0.886
**rs6017218**	42555737	20	qio2	G	1.168	0.155	0.136	0.252
**rs6017218**	42555737	20	rad3	G	0.824	-0.193	0.076	0.011 *
**rs6017218**	42555737	20	rage	G	1.020	0.020	0.208	0.924
**rs6017218**	42555737	20	rai2	G	0.903	-0.102	0.265	0.699
**rs6017218**	42555737	20	rau2	G	1.455	0.375	0.188	0.046 *
**rs6017218**	42555737	20	rde4	G	1.382	0.324	0.212	0.127
**rs6017218**	42555737	20	rot4	G	0.770	-0.262	0.164	0.111
**rs6017218**	42555737	20	twg2	G	1.092	0.088	0.076	0.248
**rs6031242**	42556096	20	boma	G	1.033	0.032	0.122	0.791
**rs6031242**	42556096	20	cof3	G	0.638	-0.449	0.296	0.129
**rs6031242**	42556096	20	gep3	G	0.963	-0.038	0.185	0.838
**rs6031242**	42556096	20	grdg	G	0.918	-0.086	0.148	0.564
**rs6031242**	42556096	20	grnd	G	0.938	-0.064	0.137	0.643
**rs6031242**	42556096	20	gsk2	G	0.921	-0.082	0.105	0.435
**rs6031242**	42556096	20	i2b3	G	1.041	0.040	0.107	0.708
**rs6031242**	42556096	20	mmi2	G	1.182	0.167	0.144	0.246
**rs6031242**	42556096	20	mmo4	G	1.239	0.214	0.221	0.333
**rs6031242**	42556096	20	qi3c	G	0.950	-0.052	0.123	0.674
**rs6031242**	42556096	20	qi6c	G	1.090	0.086	0.141	0.540
**rs6031242**	42556096	20	qio2	G	1.154	0.143	0.143	0.317
**rs6031242**	42556096	20	rad3	G	0.841	-0.174	0.080	0.031 *
**rs6031242**	42556096	20	rage	G	1.057	0.055	0.214	0.796
**rs6031242**	42556096	20	rai2	G	0.854	-0.158	0.278	0.569
**rs6031242**	42556096	20	rau2	G	1.344	0.296	0.198	0.135
**rs6031242**	42556096	20	rde4	G	1.287	0.252	0.216	0.244
**rs6031242**	42556096	20	rot4	G	0.652	-0.428	0.179	0.017 *
**rs6031242**	42556096	20	twg2	G	1.075	0.072	0.079	0.362
**rs6031245**	42559531	20	boma	T	0.999	-0.001	0.137	0.997
**rs6031245**	42559531	20	cof3	T	0.626	-0.468	0.347	0.177
**rs6031245**	42559531	20	col3	T	0.901	-0.104	0.140	0.458
**rs6031245**	42559531	20	edi2	T	0.895	-0.111	0.204	0.586

rs id	Pos	Chr	Cohort	A1	OR	logOR	SE (logOR)	P
**rs6031245**	42559531	20	gep3	T	0.892	-0.115	0.215	0.592
**rs6031245**	42559531	20	grdg	T	0.964	-0.037	0.163	0.821
**rs6031245**	42559531	20	grnd	T	0.925	-0.078	0.154	0.615
**rs6031245**	42559531	20	gsk2	T	0.930	-0.073	0.122	0.549
**rs6031245**	42559531	20	i2b3	T	1.080	0.077	0.122	0.529
**rs6031245**	42559531	20	mmi2	T	0.978	-0.023	0.157	0.885
**rs6031245**	42559531	20	mmo4	T	1.259	0.230	0.249	0.355
**rs6031245**	42559531	20	nes1	T	1.050	0.049	0.092	0.597
**rs6031245**	42559531	20	qi3c	T	1.005	0.005	0.131	0.970
**rs6031245**	42559531	20	qi6c	T	1.161	0.149	0.155	0.336
**rs6031245**	42559531	20	qio2	T	1.086	0.083	0.166	0.619
**rs6031245**	42559531	20	rad3	T	0.837	-0.178	0.091	0.052
**rs6031245**	42559531	20	rage	T	1.355	0.304	0.263	0.248
**rs6031245**	42559531	20	rai2	T	0.715	-0.336	0.306	0.272
**rs6031245**	42559531	20	rau2	T	1.323	0.280	0.210	0.183
**rs6031245**	42559531	20	rde4	T	1.100	0.095	0.239	0.690
**rs6031245**	42559531	20	rot4	T	0.551	-0.596	0.225	0.008 *
**rs6031245**	42559531	20	twg2	T	1.133	0.125	0.089	0.162
**rs6093898**	42566577	20	boma	G	0.968	-0.032	0.137	0.814
**rs6093898**	42566577	20	cof3	G	0.672	-0.397	0.343	0.247
**rs6093898**	42566577	20	col3	G	0.898	-0.107	0.140	0.445
**rs6093898**	42566577	20	edi2	G	0.933	-0.069	0.204	0.735
**rs6093898**	42566577	20	gep3	G	0.884	-0.124	0.215	0.564
**rs6093898**	42566577	20	grdg	G	0.947	-0.055	0.163	0.737
**rs6093898**	42566577	20	grnd	G	0.944	-0.058	0.154	0.710
**rs6093898**	42566577	20	gsk2	G	0.923	-0.080	0.122	0.512
**rs6093898**	42566577	20	i2b3	G	1.094	0.090	0.121	0.460
**rs6093898**	42566577	20	mmi2	G	0.985	-0.015	0.157	0.922
**rs6093898**	42566577	20	mmo4	G	1.137	0.128	0.247	0.603
**rs6093898**	42566577	20	nes1	G	1.041	0.040	0.090	0.657
**rs6093898**	42566577	20	qi3c	G	1.027	0.027	0.135	0.844
**rs6093898**	42566577	20	qi6c	G	1.161	0.149	0.155	0.335
**rs6093898**	42566577	20	qio2	G	1.129	0.121	0.164	0.460
**rs6093898**	42566577	20	rad3	G	0.838	-0.176	0.091	0.053
**rs6093898**	42566577	20	rage	G	1.371	0.316	0.262	0.228
**rs6093898**	42566577	20	rai2	G	0.715	-0.336	0.306	0.272
**rs6093898**	42566577	20	rau2	G	1.265	0.235	0.208	0.258
**rs6093898**	42566577	20	rde4	G	1.069	0.067	0.234	0.776
**rs6093898**	42566577	20	rot4	G	0.579	-0.546	0.222	0.014 *
**rs6093898**	42566577	20	twg2	G	1.131	0.123	0.089	0.167
**rs4812767**	42568829	20	boma	T	0.973	-0.027	0.138	0.845
**rs4812767**	42568829	20	cof3	T	0.620	-0.478	0.347	0.168
**rs4812767**	42568829	20	col3	T	0.901	-0.104	0.140	0.459

rs id	Pos	Chr	Cohort	A1	OR	logOR	SE (logOR)	P
**rs4812767**	42568829	20	edi2	T	0.924	-0.079	0.204	0.700
**rs4812767**	42568829	20	gep3	T	0.889	-0.117	0.214	0.584
**rs4812767**	42568829	20	grdg	T	0.954	-0.047	0.163	0.774
**rs4812767**	42568829	20	grnd	T	0.896	-0.110	0.155	0.480
**rs4812767**	42568829	20	gsk2	T	0.930	-0.073	0.122	0.549
**rs4812767**	42568829	20	i2b3	T	1.098	0.093	0.122	0.444
**rs4812767**	42568829	20	mmi2	T	0.968	-0.033	0.158	0.835
**rs4812767**	42568829	20	mmo4	T	1.336	0.290	0.251	0.249
**rs4812767**	42568829	20	nes1	T	1.047	0.046	0.092	0.619
**rs4812767**	42568829	20	qi3c	T	1.023	0.023	0.133	0.865
**rs4812767**	42568829	20	qi6c	T	1.161	0.149	0.155	0.335
**rs4812767**	42568829	20	qio2	T	1.128	0.120	0.165	0.467
**rs4812767**	42568829	20	rad3	T	0.827	-0.190	0.092	0.038 *
**rs4812767**	42568829	20	rage	T	1.371	0.316	0.262	0.228
**rs4812767**	42568829	20	rai2	T	0.728	-0.317	0.306	0.300
**rs4812767**	42568829	20	rau2	T	1.283	0.249	0.208	0.232
**rs4812767**	42568829	20	rde4	T	1.069	0.067	0.234	0.776
**rs4812767**	42568829	20	rot4	T	0.552	-0.595	0.225	0.008 *
**rs4812767**	42568829	20	twg2	T	1.144	0.135	0.089	0.133

Table S13. Meta-analysis results for five SNPs in groups and combined sample of PGC and all UK replication samples.

rs id	Group	OR	logOR	SE (logOR)	Lower (OR)	Upper (OR)	p
**rs4812767**	PGC_AUS	1.057	0.056	0.084	0.897	1.246	0.507
**rs4812767**	PGC_GER	1.008	0.008	0.072	0.875	1.162	0.912
**rs4812767**	PGC_MIXED	0.943	-0.059	0.120	0.745	1.194	0.625
**rs4812767**	PGC_NET	0.955	-0.046	0.085	0.808	1.129	0.590
**rs4812767**	PGC_SWE	1.144	0.135	0.089	0.960	1.363	0.133
**rs4812767**	PGC_UK_IRE	0.840	-0.174	0.075	0.725	0.974	0.021*
**rs4812767**	PGC_USA	1.034	0.034	0.077	0.889	1.202	0.662
**rs4812767**	UKB+PGC_UK_IRE	0.938	-0.064	0.032	0.881	0.998	0.044*
**rs4812767**	PGC_combined22	1.002	0.002	0.029	0.946	1.061	0.956
**rs6017218**	PGC_AUS	1.016	0.016	0.070	0.886	1.166	0.817
**rs6017218**	PGC_GER	1.013	0.013	0.061	0.899	1.142	0.827
**rs6017218**	PGC_MIXED	1.382	0.324	0.212	0.912	2.095	0.127
**rs6017218**	PGC_NET	0.770	-0.262	0.164	0.558	1.062	0.111
**rs6017218**	PGC_SWE	1.092	0.088	0.076	0.941	1.268	0.248
**rs6017218**	PGC_UK_IRE	0.842	-0.172	0.068	0.737	0.961	0.011*
**rs6017218**	PGC_USA	1.087	0.084	0.065	0.958	1.234	0.197
**rs6017218**	UKB+PGC_UK_IRE	0.928	-0.074	0.028	0.880	0.980	0.007*
**rs6017218**	PGC_combined22	1.002	0.002	0.031	0.946	1.061	0.956
**rs6031242**	PGC_AUS	1.016	0.016	0.075	0.877	1.177	0.835
**rs6031242**	PGC_GER	1.034	0.034	0.064	0.913	1.171	0.596
**rs6031242**	PGC_MIXED	1.287	0.252	0.216	0.842	1.967	0.244
**rs6031242**	PGC_NET	0.652	-0.428	0.179	0.458	0.926	0.017*
**rs6031242**	PGC_SWE	1.075	0.072	0.079	0.920	1.256	0.362
**rs6031242**	PGC_UK_IRE	0.858	-0.153	0.071	0.747	0.987	0.032*
**rs6031242**	PGC_USA	1.018	0.018	0.069	0.890	1.165	0.794
**rs6031242**	UKB+PGC_UK_IRE	0.932	-0.070	0.029	0.881	0.987	0.016*
**rs6031242**	PGC_combined22	0.989	-0.011	0.031	0.931	1.051	0.720
**rs6031245**	PGC_AUS	1.040	0.039	0.083	0.883	1.224	0.642
**rs6031245**	PGC_GER	1.012	0.012	0.072	0.878	1.166	0.872
**rs6031245**	PGC_MIXED	0.949	-0.053	0.121	0.748	1.202	0.662
**rs6031245**	PGC_NET	0.957	-0.044	0.085	0.810	1.131	0.607
**rs6031245**	PGC_SWE	1.133	0.125	0.089	0.951	1.350	0.162
**rs6031245**	PGC_UK_IRE	0.843	-0.171	0.075	0.727	0.977	0.024*
**rs6031245**	PGC_USA	1.041	0.040	0.077	0.895	1.211	0.600
**rs6031245**	UKB+PGC_UK_IRE	0.937	-0.066	0.032	0.880	0.997	0.040*
**rs6031245**	PGC_combined22	0.991	-0.009	0.031	0.932	1.054	0.767
**rs6093898**	PGC_AUS	1.064	0.062	0.084	0.903	1.254	0.460
**rs6093898**	PGC_GER	0.995	-0.005	0.072	0.863	1.146	0.941
**rs6093898**	PGC_MIXED	0.941	-0.061	0.120	0.743	1.191	0.611
**rs6093898**	PGC_NET	0.957	-0.044	0.084	0.812	1.128	0.603

rs id	Group	OR	logOR	SE (logOR)	Lower (OR)	Upper (OR)	p
**rs6093898**	PGC_SWE	1.131	0.123	0.089	0.950	1.347	0.167
**rs6093898**	PGC_UK_IRE	0.848	-0.165	0.075	0.732	0.983	0.028*
**rs6093898**	PGC_USA	1.042	0.041	0.077	0.897	1.211	0.589
**rs6093898**	UKB+PGC_UK_IRE	0.937	-0.065	0.032	0.881	0.998	0.042*
**rs6093898**	PGC_combined22	0.991	-0.009	0.031	0.932	1.054	0.772

Table S14. Genes showing similar expression patterns with RP1-269M15.3 (r≥0.7) in development brain tissues in BRAINSPAN. r: correlation of gene expression with RP1-269M15.3. The data was downloaded from BrainSpan: Atlas of the Developing Human Brain [Internet]. Funded by ARRA Awards 1RC2MH089921-01, 1RC2MH090047-01, and 1RC2MH089929-01. © 2011. Available from: http://www.brainspan.org/.

Gene Symbol	Chromosome	r
**RP1-269M15.3**	20	1
**PTPRT**	20	0.833
**LRFN5**	14	0.78
**GRM7**	3	0.77
**EPHA10**	1	0.746
**RP11-497E19.1**	14	0.739
**RP11-586D19.1**	4	0.737
**FAM78B**	1	0.736
**RP11-497E19.2**	14	0.736
**DLEU7**	13	0.733
**C9orf91**	9	0.73
**CACNA2D3**	3	0.72
**FBXL2**	3	0.716
**LRRC4C**	11	0.716
**FAM84A**	2	0.711
**RFPL1-AS1**	22	0.711
**DCBLD1**	6	0.709
**RFPL1**	22	0.709
**CHSY3**	5	0.705
**PPFIA2**	12	0.704
**ARMCX2**	X	0.701
**RP11-133K1.2**	15	0.7

Table S15. Genes showing similar expression patterns with *TOX2* (r≥0.7) in development brain tissues in BRAINSPAN. r: correlation of gene expression with *TOX2*. The data was downloaded from BrainSpan: Atlas of the Developing Human Brain [Internet]. Funded by ARRA Awards 1RC2MH089921-01, 1RC2MH090047-01, and 1RC2MH089929-01. © 2011. Available from: http://www.brainspan.org/.

Gene Symbol	Chromosome	r
**TOX2**	20	1
**GPR123**	10	0.808
**KIF17**	1	0.761
**CRH**	8	0.754
**SYT17**	16	0.748
**VWC2L**	2	0.744
**ARID5B**	10	0.73
**CPNE7**	16	0.73
**NECAB2**	16	0.719
**NTNG1**	1	0.716
**STBD1**	4	0.713
**CYB561**	17	0.707
**FAM127A**	X	0.705
**RP13-137A17.4**	10	0.702

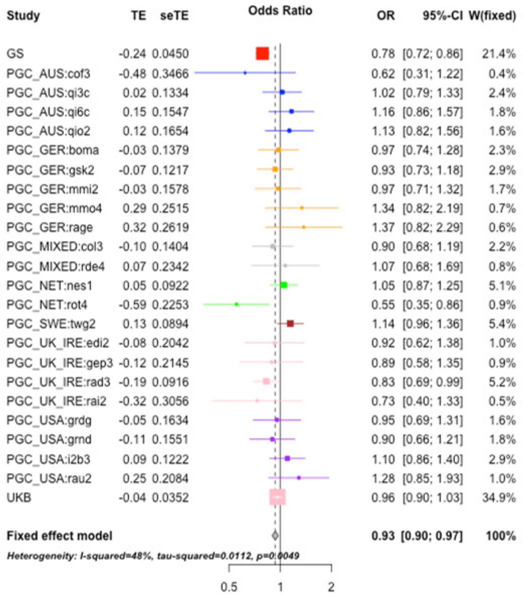

Figure S1A. Forest plots of meta-analysis of GS:SFHS, PGC2-MDD individual cohorts and UK Biobank for rs4812767.

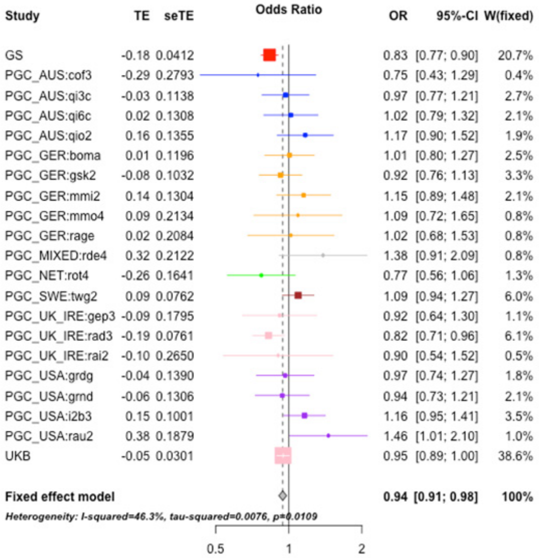

Figure S1B. Forest plots of meta-analysis of GS:SFHS, PGC2-MDD individual cohorts and UK Biobank for rs6017218.

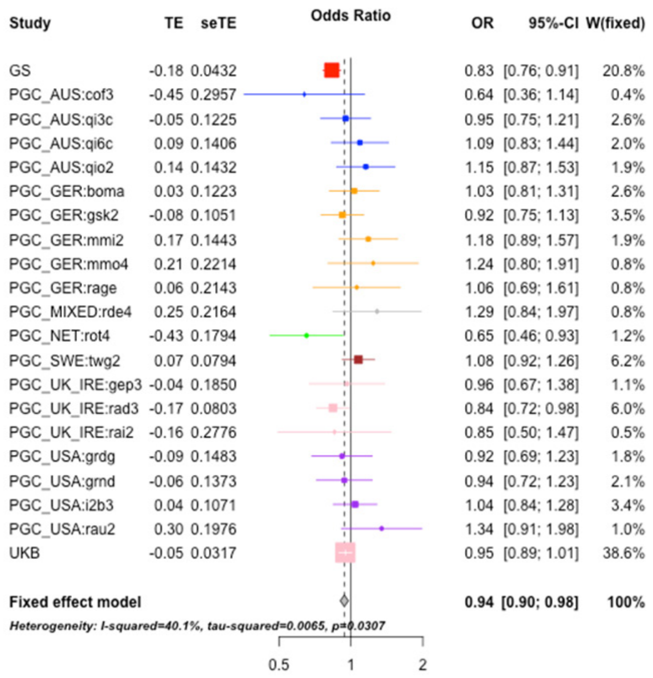

Figure S1C. Forest plots of meta-analysis of GS:SFHS, PGC2-MDD individual cohorts and UK Biobank for rs6031242.

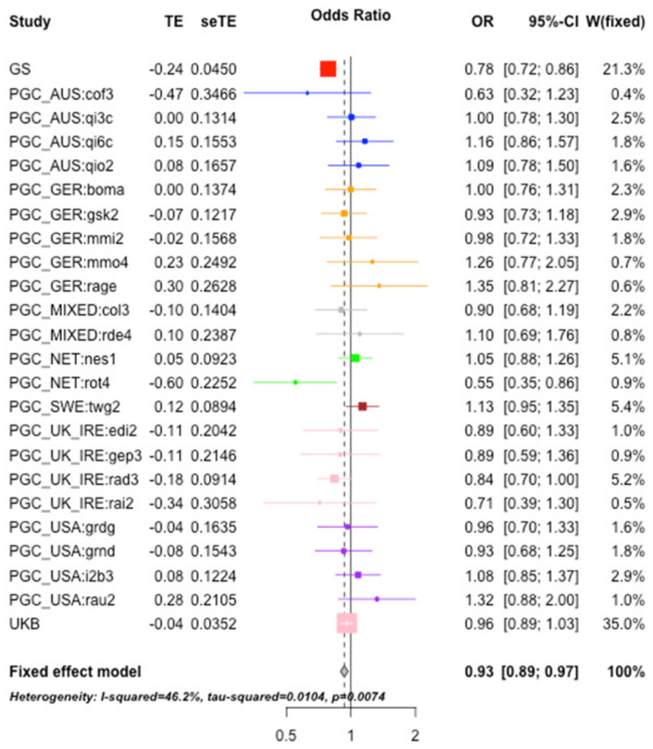

Figure S1D. Forest plots of meta-analysis of GS:SFHS, PGC2-MDD individual cohorts and UK Biobank for rs6031245.

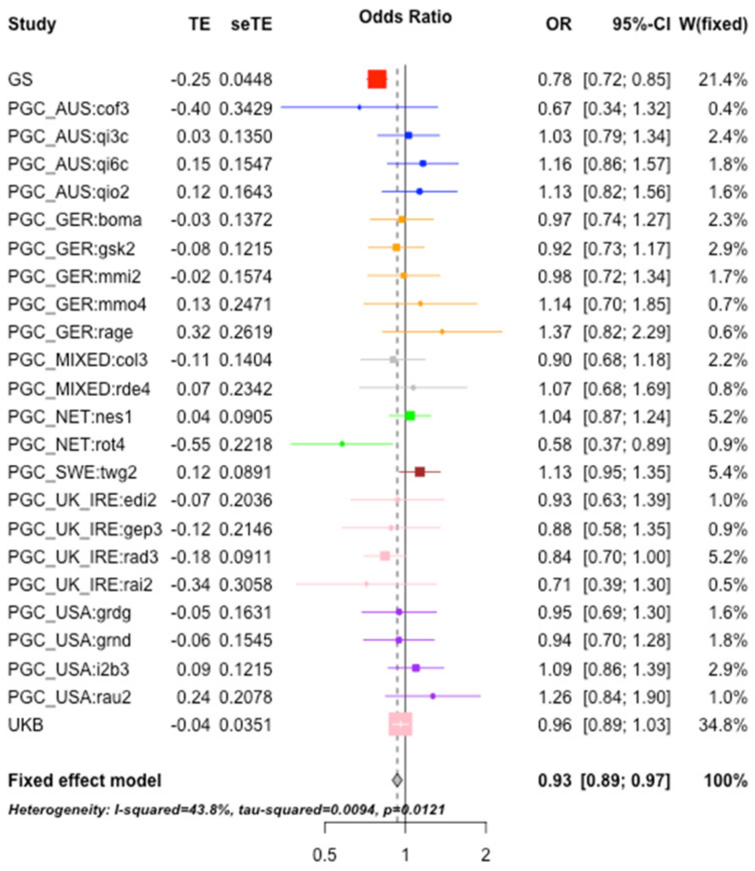

Figure S1E. Forest plots of meta-analysis of GS:SFHS, PGC2-MDD individual cohorts and UK Biobank for rs6093898.

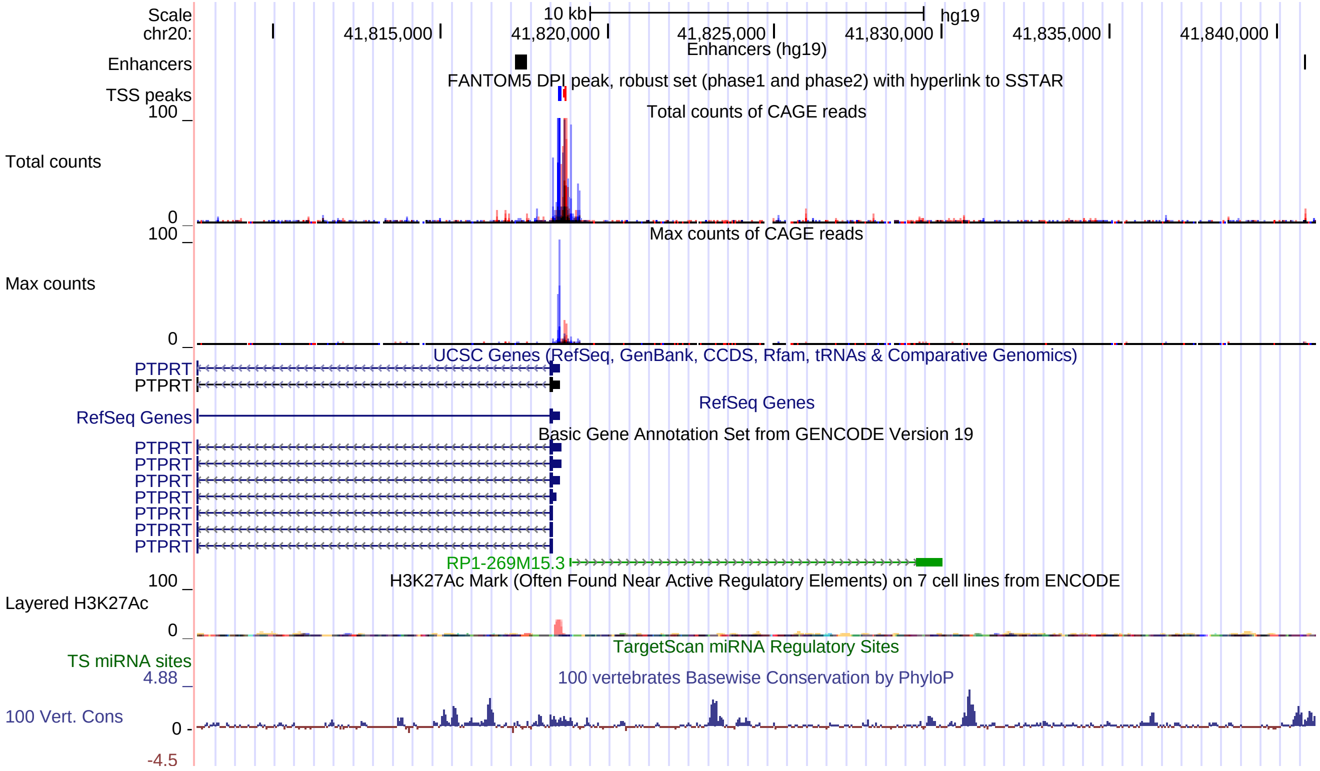

Figure S2A. Functional annotation for RP1-269M15.3.

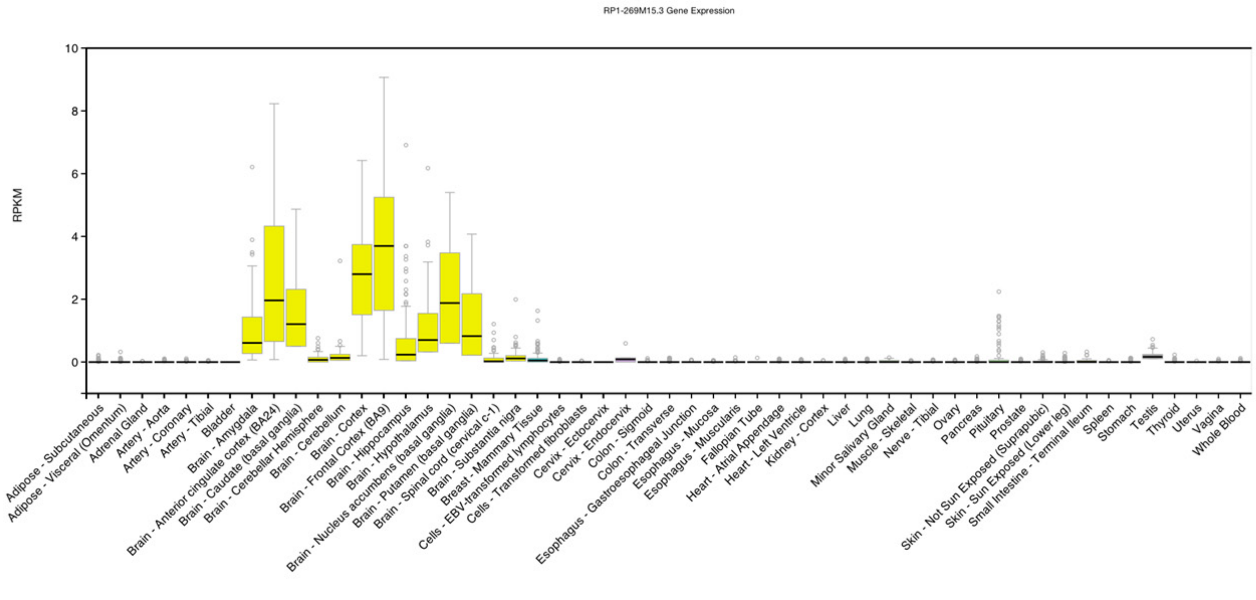

Figure S2B. Gene expression pattern of RP1-269M15.3 across different tissues (downloaded from http://www.gtexportal.org/home/gene/RP1-269M15.3).

Supplemental References

1. Gunderson KL (2009) Whole-genome genotyping on bead arrays. Methods Mol Biol 529: 197-213.
2. Smith BH, Campbell H, Blackwood D, Connell J, Connor M, Deary IJ, et al. (2006) Generation Scotland: the Scottish Family Health Study; a new resource for researching genes and heritability. BMC Med Genet 7: 74.
3. Delaneau O, Marchini J, Zagury JF (2012) A linear complexity phasing method for thousands of genomes. Nat Methods 9: 179-181.
4. First MB, Spitzer RL, Gibbon M, Williams JB (2001) Structured clinical interview for DSM-IV-TR axis I disorders—non-patient edition. New York State Psychiatric Institute, New York.
5. Fernandez-Pujals AM, Adams MJ, Thomson P, McKechanie AG, Blackwood DH, Smith BH, et al. (2015) Epidemiology and Heritability of Major Depressive Disorder, Stratified by Age of Onset, Sex, and Illness Course in Generation Scotland: Scottish Family Health Study (GS:SFHS). PLoS One 10: e0142197.
6. Sudlow C, Gallacher J, Allen N, Beral V, Burton P, Danesh J, et al. (2015) UK biobank: an open access resource for identifying the causes of a wide range of complex diseases of middle and old age. PLoS Med 12: e1001779.
7. Howie B, Marchini J, Stephens M (2011) Genotype Imputation with Thousands of Genomes. G3-Genes Genomes Genetics 1: 457-469.
8. Yang J, Bakshi A, Zhu Z, Hemani G, Vinkhuyzen AAE, Nolte IM, et al. (2015) Genome-wide genetic homogeneity between sexes and populations for human height and body mass index. Human Molecular Genetics 24: 7445-7449.
9. Smith DJ, Nicholl BI, Cullen B, Martin D, Ul-Haq Z, Evans J, et al. (2013) Prevalence and characteristics of probable major depression and bipolar disorder within UK biobank: cross-sectional study of 172,751 participants. PLoS One 8: e75362.
10. Lee SH, Ripke S, Neale BM, Faraone SV, Purcell SM, Perlis RH, et al. (2013) Genetic relationship between five psychiatric disorders estimated from genome-wide SNPs. Nature Genetics 45: 984-+.
11. Zeng Y, Navarro P, Fernandez-Pujals AM, Hall LS, Clarke T-K, Thomson PA, et al. (2016) A Combined Pathway and Regional Heritability Analysis Indicates NETRIN1 Pathway is Associated with Major Depressive Disorder. Biological Psychiatry.
12. Yang J, Lee SH, Goddard ME, Visscher PM (2011) GCTA: a tool for genome-wide complex trait analysis. Am J Hum Genet 88: 76-82.
13. Aulchenko YS, Ripke S, Isaacs A, Van Duijn CM (2007) GenABEL: an R library for genome-wide association analysis. Bioinformatics 23: 1294-1296.
14. Nagamine Y, Pong-Wong R, Navarro P, Vitart V, Hayward C, Rudan I, et al. (2012) Localising loci underlying complex trait variation using Regional Genomic Relationship Mapping. PLoS One 7: e46501.
15. Shirali M, Pong-Wong R, Navarro P, Knott S, Hayward C, Vitart V, et al. (2016) Regional heritability mapping method helps explain missing heritability of blood lipid traits in isolated populations. Heredity (Edinb) 116: 333-338.
16. Cebamanos L, Gray A, Stewart I, Tenesa A (2014) Regional heritability advanced complex trait analysis for GPU and traditional parallel architectures. Bioinformatics 30: 1177-1179.
17. Wang M, Lin SL (2014) FamLBL: detecting rare haplotype disease association based on common SNPs using case-parent triads. Bioinformatics 30: 2611-2618.
18. Boyle AP, Hong EL, Hariharan M, Cheng Y, Schaub MA, Kasowski M, et al. (2012) Annotation of functional variation in personal genomes using RegulomeDB. Genome Res 22: 1790-1797.
19. Ritchie GR, Dunham I, Zeggini E, Flicek P (2014) Functional annotation of noncoding sequence variants. Nat Methods 11: 294-296.

20. Davydov EV, Goode DL, Sirota M, Cooper GM, Sidow A, Batzoglou S (2010) Identifying a High Fraction of the Human Genome to be under Selective Constraint Using GERP plus. Plos Computational Biology 6.

21. Dabney A, Storey JD, Warnes G (2010) qvalue: Q-value estimation for false discovery rate control. R package version 1.

22. Jaffe AE, Gao Y, Deep-Soboslay A, Tao R, Hyde TM, Weinberger DR, et al. (2016) Mapping DNA methylation across development, genotype and schizophrenia in the human frontal cortex. Nat Neurosci 19: 40-47.
